# An Ovol2-Zeb1 Mutual Inhibitory Circuit Governs Bidirectional and Multi-step Transition between Epithelial and Mesenchymal States

**DOI:** 10.1371/journal.pcbi.1004569

**Published:** 2015-11-10

**Authors:** Tian Hong, Kazuhide Watanabe, Catherine Ha Ta, Alvaro Villarreal-Ponce, Qing Nie, Xing Dai

**Affiliations:** 1 Department of Mathematics, University of California, Irvine, Irvine, California, United States of America; 2 Center for Complex Biological Systems, University of California, Irvine, Irvine, California, United States of America; 3 Department of Biological Chemistry, School of Medicine, University of California, Irvine, Irvine, California, United States of America; Imperial College London, UNITED KINGDOM

## Abstract

Reversible epithelial-to-mesenchymal transition (EMT) is central to tissue development, epithelial stemness, and cancer metastasis. While many regulatory elements have been identified to induce EMT, the complex process underlying such cellular plasticity remains poorly understood. Utilizing a systems biology approach integrating modeling and experiments, we found multiple intermediate states contributing to EMT and that the robustness of the transitions is modulated by transcriptional factor Ovol2. In particular, we obtained evidence for a mutual inhibition relationship between Ovol2 and EMT inducer Zeb1, and observed that adding this regulation generates a novel four-state system consisting of two distinct intermediate phenotypes that differ in differentiation propensities and are favored in different environmental conditions. We identified epithelial cells that naturally exist in an intermediate state with bidirectional differentiation potential, and found the balance between EMT-promoting and -inhibiting factors to be critical in achieving and selecting between intermediate states. Our analysis suggests a new design principle in controlling cellular plasticity through multiple intermediate cell fates and underscores the critical involvement of Ovol2 and its associated molecular regulations.

## Author Summary

Cumulative evidence reveals remarkable lineage plasticity of somatic cells. Epithelial-to-mesenchymal transition (EMT) represents a prime example of such plasticity where an epithelial cell is converted into a mesenchymal cell. This process is used in normal development to generate crucial cell types, and is hijacked by cancer cells for invasion and metastasis. Recent studies also suggest the importance of EMT in generating stem cell properties. The reversibility of EMT and its sensitivity to varying environmental stimuli pose interesting challenges to understand the intricate regulatory networks that direct cellular state transitions and their dynamics. Here we use a systems biology approach to probe into the complexity of the EMT process. We report a new molecular regulation that expands the known regulatory network, and show that this new network is capable of generating multiple intermediate states, which we provide experimental evidence for. We present modeling and experimental results to highlight the significance of a delicate balance between EMT-promoting and -inhibiting factors for achieving and/or selecting an intermediate state, and to suggest the biological significance of the multiple intermediate states. This work further elucidates the complex strategies that control epithelial cell behavior and cancer/stem cell plasticity.

## Introduction

Epithelial-to-mesenchymal transition (EMT) represents an extreme form of cellular plasticity where an epithelial cell is converted into a mesenchymal cell. Complete EMT is essential during embryogenesis to generate crucial developmental cell types [1], whereas partial EMT occurs in committed epithelial tissues with yet unknown functional significance [2]. Recently, EMT has been shown to promote stem cell properties, as differentiated epithelial cells that have undergone a round of EMT gain multipotency and self-renewal capability [3–5]. Furthermore, reversible EMT plays important roles in pathological processes such as cancer metastasis and wound healing. EMT endows cancer cells with the ability to migrate and invade adjacent tissues through changes in adhesion and behavior. Upon arrival to the destination site, EMTed cancer cells can revert to the epithelial phenotype via mesenchymal-to-epithelial transition (MET) to settle and differentiate into secondary tumors [1].

Previous studies have identified key transcription factors (TFs) and microRNAs (miRNAs) that are involved in the regulation of EMT. In particular, mutual inhibition loops formed between Zeb1 and miR-200 [6], and between Snail and miR-34a [7] are critical components in the regulatory network [8]. Mathematical modeling suggested that these mutual inhibition loops govern a tri-stable system, in which cells can be stabilized at an epithelial (E) state, a mesenchymal (M) state, or an intermediate state exhibiting expression of signature genes of both E and M in a variable fashion [9,10]. The intermediate state identified by these models is proposed to associate with cancer cells that exhibit collective migration during tumorigenesis [9], implicating the clinical relevance of the ternary switch in cell plasticity.

In recent experimental studies, we showed that transcription factor Ovol2 restricts EMT by directly inhibiting EMT-inducing factors including Zeb1, and that these regulations are critical for proper morphogenesis and for maintaining epithelial lineages in mammary gland and skin epidermis [11,12]. However, the precise role of Ovol2 in the context of the well-studied core molecular network that controls EMT dynamics remains to be elucidated. In addition, it is unclear how EMT-inhibiting transcriptional factors like Ovol2 and EMT-promoting transcription factors like Zeb1 interact integratively to regulate the intermediate state.

In this work, we first provide new experimental evidence suggesting a direct regulation of Ovol2 by Zeb1, which together with previous reports of Ovol2 inhibition of Zeb1 [11–13] demonstrates the existence of an Ovol2-Zeb1 mutual inhibition circuit. We then present a mathematical model that includes this new regulation, revealing two, rather than one, intermediate states with distinct propensities to differentiate into E and M states. We show that the Ovol2-Zeb1 mutual inhibition circuit is essential for the existence and robustness of both intermediate states in this model, and experimentally validate a specific prediction of the model, namely that Ovol2 is able to reprogram any given states to an E state. Furthermore, we describe experimental results suggesting that mammary epithelial cell line MCF10A represents one of the intermediate states that exhibit a bidirectional potential to differentiate into both E and M states. Together, our findings uncover a new layer of complexity of the dynamic, multi-step transitions between E and M states and unravel key regulatory mechanisms that control such transitions.

## Results

### A regulatory network containing Ovol2-Zeb1 mutual repression results in multiple intermediate states and a four-state EMT system

**Fig 1:**
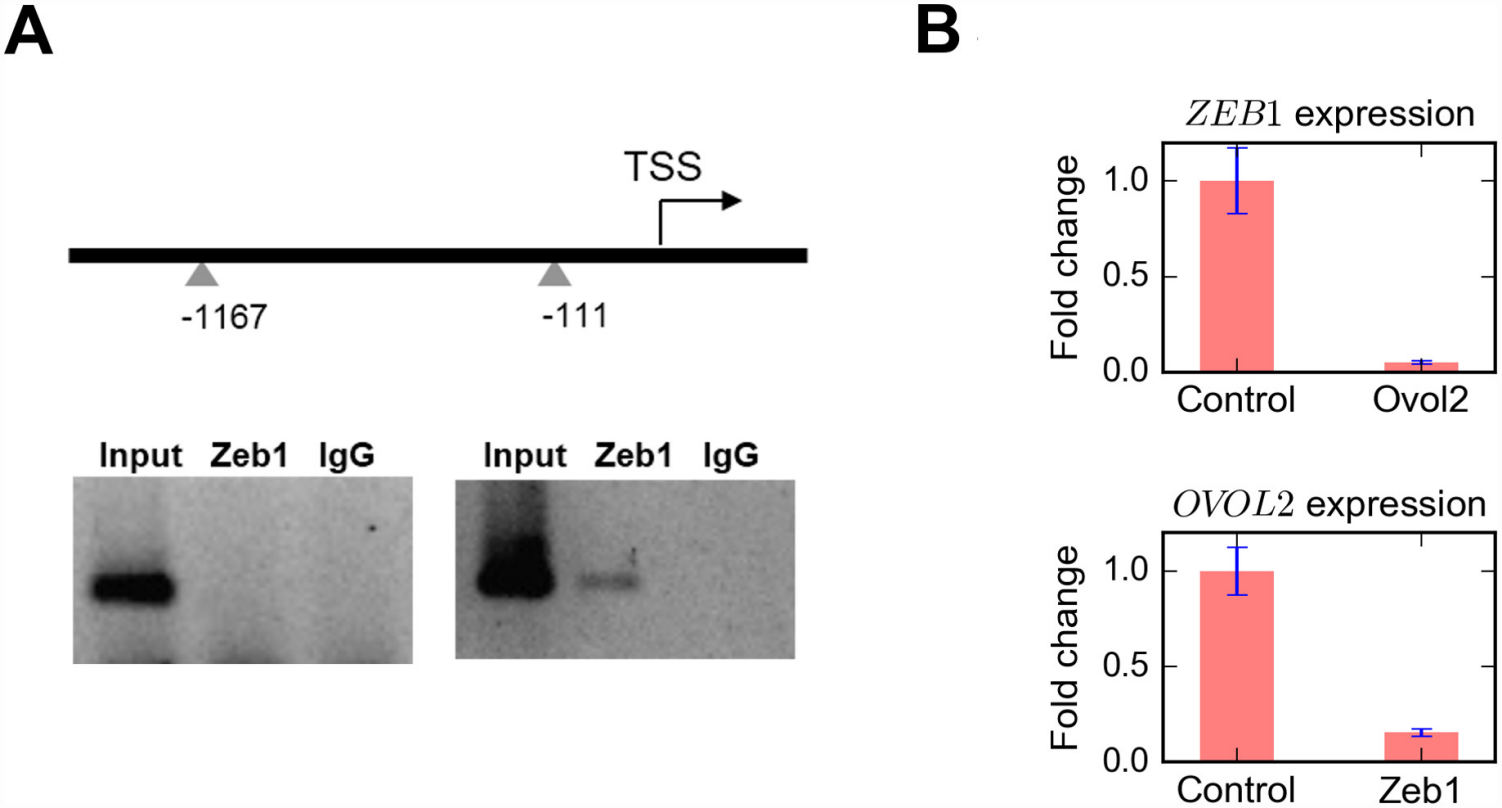
Experimental evidence for Ovol2-Zeb1 mutual repression. **A)** Zeb1 binds to the *Ovol2* promoter in mouse mammary epithelial cells. Top, diagram showing the presence of Zeb1 consensus motifs (triangles) in *Ovol2* promoter. Bottom, ChIP-PCR using primers for the upstream (left) and downstream sites (right). **B)** RT-quantitative PCR analysis showing that OVOL2 and ZEB1 overexpression in MCF10A cells results in decreased level of *Zeb1* and *Ovol2* transcripts, respectively TSS, transcription start site.

Mutual inhibition loops between EMT-inducing TFs and miRNAs (e.g. Zeb1-miR200 and Snail-miR34a) are critical for robust control of EMT/MET [8]. Our previous studies showed that Zeb1 is directly inhibited by Ovol2 in mammary and skin epithelial cells [11,12]. Zeb1 and Ovol2 are expressed in a mutually exclusive pattern in clinical and cell line samples [11,13], raising the possibility that Zeb1 may also inhibit Ovol2 expression. Indeed, sequence analysis revealed the presence of two conserved Zeb1 binding consensus sequences in the human and mouse *OVOL2/Ovol2* promoters, one near the transcriptional start site (-335 bp and -111 bp for human and mouse genes, respectively) and the other further upstream (-1546 bp and -1167 bp for human and mouse, respectively) (Fig 1A). Using chromatin immunoprecipitation (ChIP) assay, we detected Zeb1 binding to the downstream but not upstream site (Fig 1A). Furthermore, forced expression of Zeb1 in MCF10A human mammary epithelial cells significantly decreased *OVOL2* expression at a transcriptional level, whereas Ovol2 overexpression led to reduced level of *ZEB1* transcript as expected (Fig 1B). These results are consistent with direct repression of *OVOL2/Ovol2* expression by Zeb1, and together with previously published data suggest the existence of an Ovol2-Zeb1 mutual inhibition loop.

**Fig 2:**
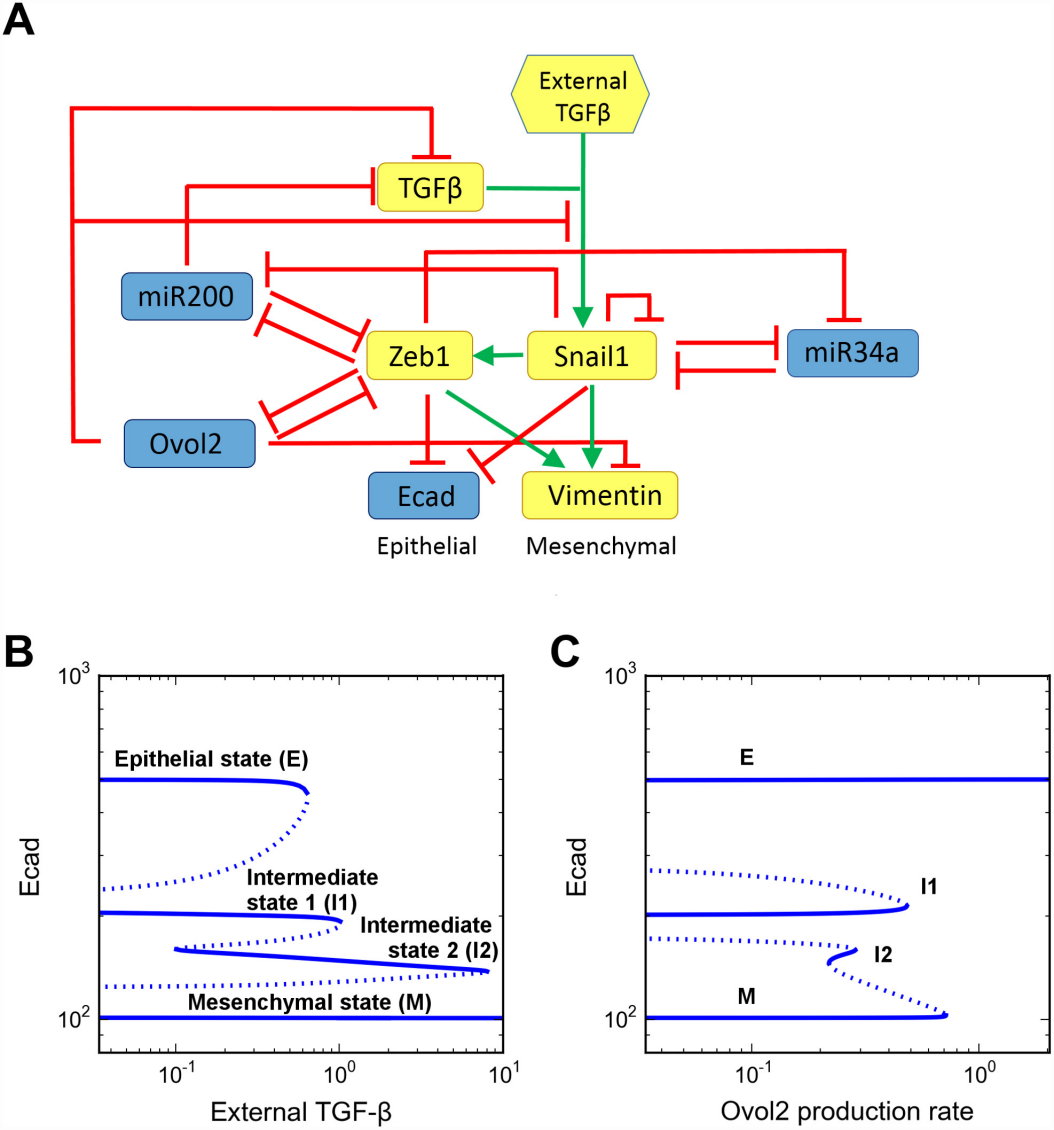
Incorporation of an Ovol2-Zeb1 mutual repression module results in the observation of four distinct states in EMT. **A)** Influence diagram of the EMT/MET system. Blue icon: epithelial promoting factor. Yellow icon: mesenchymal promoting factor. Hexagon: extracellular input. **B, C)** One-parameter bifurcation diagrams of E-cadherin (Ecad) with respect to external TGF-β (B) and Ovol2 basal production rate (C). Solid curve: stable steady state. Dashed curve: unstable steady state. In B, only transition between I1 and I2 is reversible when TGF-β level is varied. In C, varying Ovol2 alone does not result in any reversible transition, but it can possibly reverse the following TGF-β-induced transitions: E-I1, E-I2 and E-M.

To dissect the role of the Ovol2-Zeb1 loop in EMT dynamics, we incorporated this regulation, as well as the negative regulation of TGF-β signaling by Ovol2 [11] into a framework that has been successfully used to formulate a 3-state EMT system [14]. The new model thus contains three mutual inhibition loops: Zeb1-miR200, Snail-miR34a and Ovol2-Zeb1 (Fig 2A). To examine how the system might be stabilized at various stages of EMT, we performed bifurcation analysis with respect to external TGF-β as an EMT inducer. Interestingly, four distinct stable steady states corresponding to four cell phenotypes emerged with the addition of the Ovol2-Zeb1 loop (Fig 2B). In particular, two intermediate states appeared between a terminal E state and an M state (Fig 2B). We named the intermediate state closer to the E state I1, and the one closer to the M state I2. The dynamic feature of the four-state system is consistent with the recently proposed sequential cell-state transition in which more than one intermediate states may exist [15], and it is also compatible with existing EMT models [9,10,14] in terms of possible ternary switch in the system (I1-I2-M, E-I1-I2, E-I1-M or E-I2-M, depending on specific external stimulations). Importantly, our model predicted that elevated production of Ovol2 is able to reprogram all other states to the terminal E state (Fig 2C). We will return to this notion later.

### Experimental evidence supports the 4-state model and validates the reprogramming ability of Ovol2

**Fig 3:**
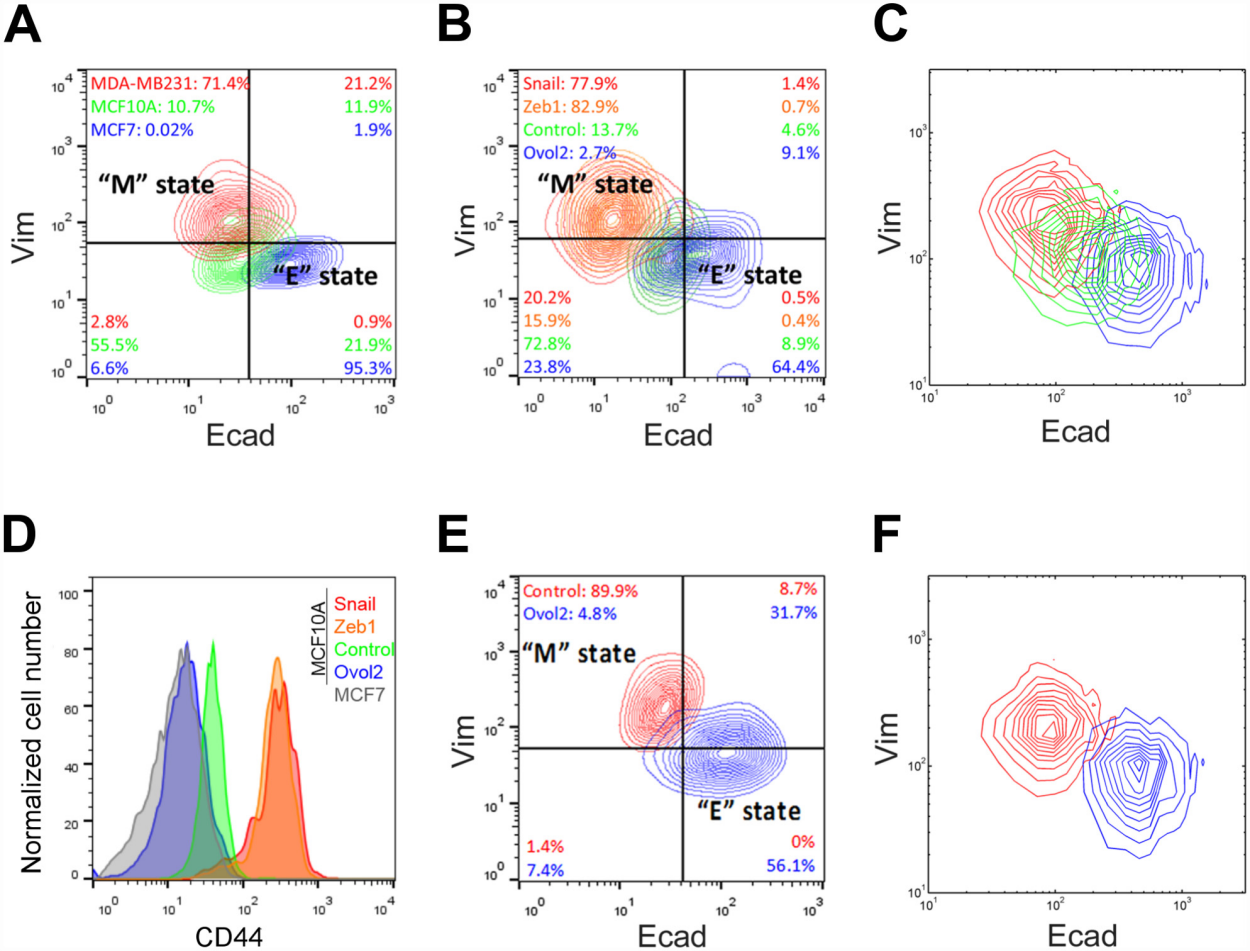
Experimental evidence for bidirectional potential of MCF10A cells. **A, B, D, E)** Flow cytometric analysis of epithelial marker (Ecad) and mesenchymal marker (Vim) profiles. **A)** Direct comparison of MCF10A with luminal (epithelial)-type cancer cell line MCF7 and basal (mesenchymal)-type cancer cell line MDA-MB231. MCF10A (green) falls in the intermediate state between MCF7 (blue) and MDA-MB231 (red). Analyses were performed at 90–100% confluency. **B)** Bidirectional potential of MCF10A cells. E(I)MT and M(I)ET was induced by forced expression of transcription factors Snail or Zeb1, and Ovol2, respectively. After 6 days of lentiviral infection, Snail (red) and Zeb1 (orange) induced EMT while Ovol2 (blue) induced MET as compared to the empty vector control (green). **C)** Stochastic simulations for a population of 2000 cells in three different conditions: basal parameter set (green), high basal production rate of Zeb1 *ZEB1* (red, *Zeb1* mRNA basal production rate was raised to 0.01 μM/hr) and high basal production rate of Ovol2 (blue, Ovol2 basal production rate was raised to 2 μM/hr). Initial conditions are all at I1 state. **D)** The histogram shows the expression status of CD44. “M” states (Snail;red and Zeb1;orange) correlate with high CD44 expression while cells in “E” state (Ovol2;blue) show low CD44 expression as compared to empty vector control (green). MCF7 is shown as a representative cell type in the “E” state with low CD44 expression. **E)** Ovol2 reprograms MDA-MB231 cells from M- to E- state. Cells were analyzed after 6 days of control (red) or Ovol2-expressing (blue) lentiviral infection. **F)** Stochastic simulations with a basal parameter set (red) and high basal production rate of Ovol2 (blue, Ovol2 basal production rate was raised to 2 μM/hr). Initial conditions are all at M state.

Previous work stipulates that unstimulated MCF10A cells are in an epithelial state and when stimulated by increasing concentrations of TGF-β they transition into first an intermediate (partial EMT) state and subsequently an M state [14]. However, by comparing the expression of epithelial (E-cadherin or Ecad) and mesenchymal (vimentin or Vim) markers between MCF10A and two breast cancer cell lines well-characterized for their cellular states (MCF7 = E state, MDA-MB231 = metastatic human breast cancer cells corresponding to an M state), we found MCF10A cells to be likely in a state that is intermediate between typical terminal E and M cells (Fig 3A, compare green population to others; S1 Fig). This is consistent with a recent study showing that MCF10A cells tend to collectively migrate [16], a feature that has been associated with the intermediate phenotype [9,17,18]. We surmise that the natural state of these cells is I1, because a majority of them show low to no Vim expression, suggesting more similarity to the terminal E than M cells.

To experimentally test whether Ovol2 is able to reprogram I1-state cells into an E state (Fig 2C), we overexpressed Ovol2 in MCF10A cells using a lentiviral expression system in which the Ovol2-expressing cells can be distinguished from uninfected cells by bicistronic expression of GFP (S2 Fig). This led to significantly increased expression of Ecad, and decreased expression of Vim as assessed by quantitative population analysis using flow cytometry (Fig 3B, blue population). Comparison with Ecad/Vim profiles in Fig 3A reveals the similarity between Ovol2-reprogrammed cells and terminal E cells (MCF7). In contrast, overexpression of EMT inducers Snail or Zeb1 directed MCF10A cells to an M phenotype (Fig 3B, red and orange populations). To compare these observations with our mathematical model, we performed stochastic simulations with the basal model upon fluctuations in gene/protein expression (see details in Methods). We started simulation with the initial condition at I1 state (Fig 3C, green). Remarkably, at high basal production rates of Zeb1 and Ovol2 (Fig 3C, red and blue), the simulation produced similar Ecad/Vim expression patterns of these populations as those observed in our experiments. Consistent with previous reports that EMT promotes stemness [3–5], the expression of a well-known cancer stem cell marker CD44 [19] decreased upon Ovol2-induced transition to E and increased upon Zeb1/Snail-induced transition to M (Fig 3D). These observations demonstrate the bidirectional differentiation potential of MCF10A cells towards two opposite directions (i.e., I1 to E or I1 to M) and provide evidence for the opposing roles of Ovol2 and Snail/Zeb1 in the dynamic EMT system of MCF10A cells.

**Fig 4:**
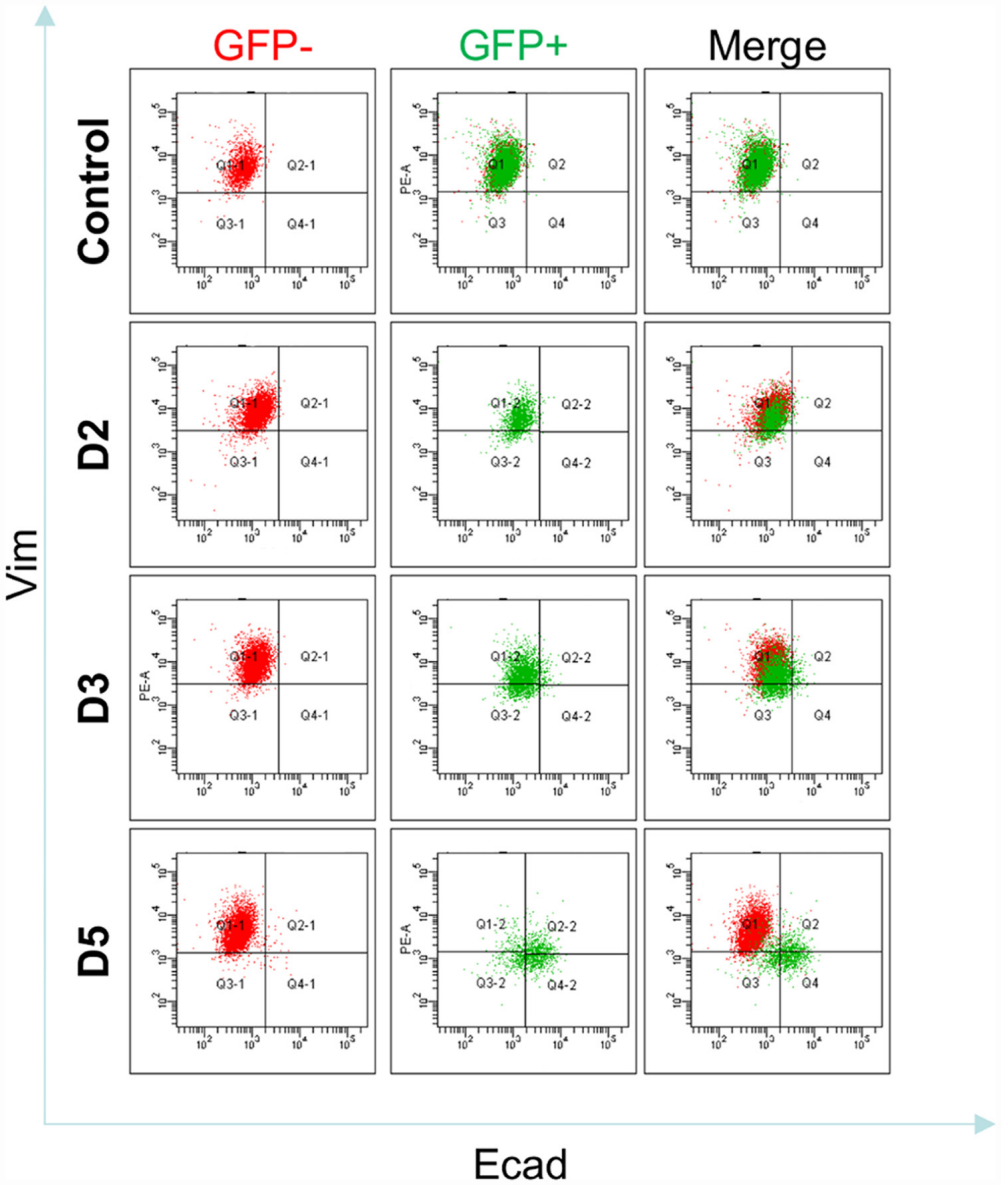
Time series of Ecad/Vim profile change upon Ovol2 expression in MDA-MB231 cells. Cells were infected with Ovol2-expressing lentivirus and Ecad/Vim profile was analyzed by flow cytometry at the indicated time points. Empty vector control at day 5 is shown at the top.

As bifurcation analysis also predicted the ability of Ovol2 to reprogram M-state cells into an E state (Fig 2C), we tested the effect of Ovol2 overexpression on MDA-MB231 cells. Indeed, forced expression of Ovol2 was able to convert these cells to exhibiting a pattern of Ecad/Vim expression that is reminiscent of the terminal E state (Fig 3E). This finding is consistent with previous reports of Ovol2 overexpression inducing epithelial features in M-state cells [11,13]. Results of stochastic simulations for control M-state cells and Ovol2-overexpressed M-state cells using the corresponding conditions are in good agreement with this observation (Fig 3F). Interestingly, a time series experiment revealed that the downregulation of Vim precedes the induction of Ecad, suggesting that upon Ovol2 expression these cells first lose their memory of the M state and then acquire the E phenotype (Fig 4).

**Fig 5:**
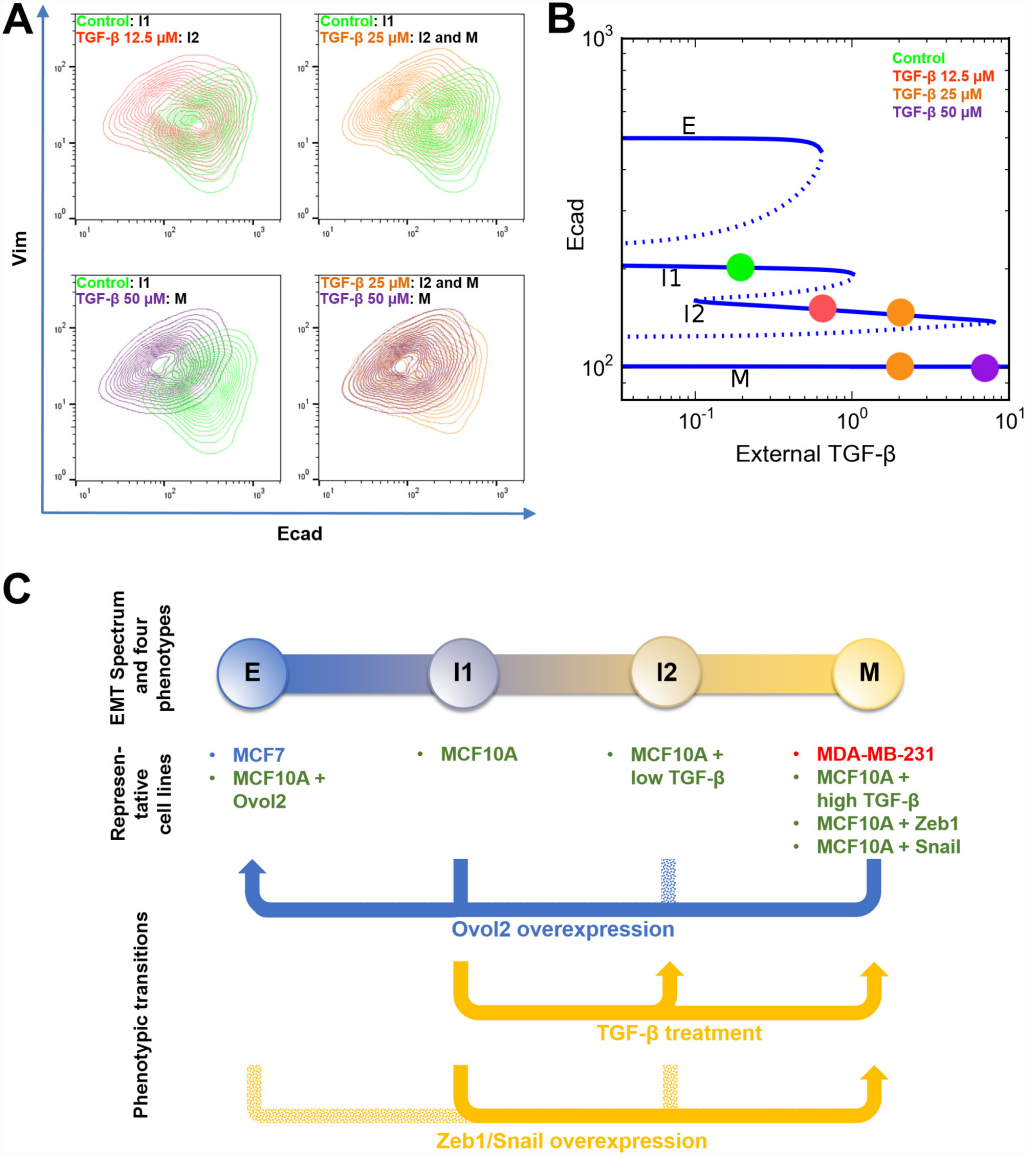
Stepwise induction of EMT in MCF10A cells by different doses of TGF-β. **A)** Cells were treated with various concentrations of TGF-β for 10 days and analyzed for Ecad/Vim expression by flow cytometry. Each panel shows a superimposed image of two treatment conditions. Note that the 25 μM TGF-β treatment gave rise to a heterogeneous population containing I2 cells and M cells (orange). **B)** The corresponding steady states of the cellular phenotypes (indicated as colored dots) observed under various TGF-β concentrations in A are mapped to the bifurcation diagram shown in Fig 2B. **C)** An illustrative summary of phenotypic transitions in the four-state system. Solid arrow represents transition with experimental evidence from this study. Dotted arrow represents hypothetical transition without experimental evidence.

Consistent with previous findings [14], a high dose of TGF-β resulted in a complete conversion of cells to what appears to be the M state, whereas low dose of TGF-β induced the appearance of two new populations in a heterogeneous culture that are likely I2 (previously termed P state for partial EMT in Zhang et al. [14]) and M states (Fig 5A and 5B and S3 Fig). Of note, in both mathematical modeling and experiments, the I2 state appears less stable than I1, as it 1) shows more vulnerability when facing fluctuations (S3 Fig); 2) entails a narrow range of Ovol2 concentration in the absence of strong TGF-β signaling (Fig 2C); and 3) is barely distinguishable from the M or I1 state experimentally and in simulations (Fig 5A and S3 Fig). The degree of separation of the different cell populations in our study is less remarkable than that reported [14], possibly due to the dynamic nature of I2 and the subtle differences in experimental conditions.

Taken together, these experimental results support our computational discovery of a four-state dynamic system. Moreover, they highlight the ability of Ovol2 in reprogramming both I1- and M-state cells into a terminal E state, as predicted by our model. The EMT phenotypes and the cell state transitions that we have discovered through modeling and experiments are summarized in Fig 5C.

### Critical regulatory controls in maintaining the four states

**Fig 6:**
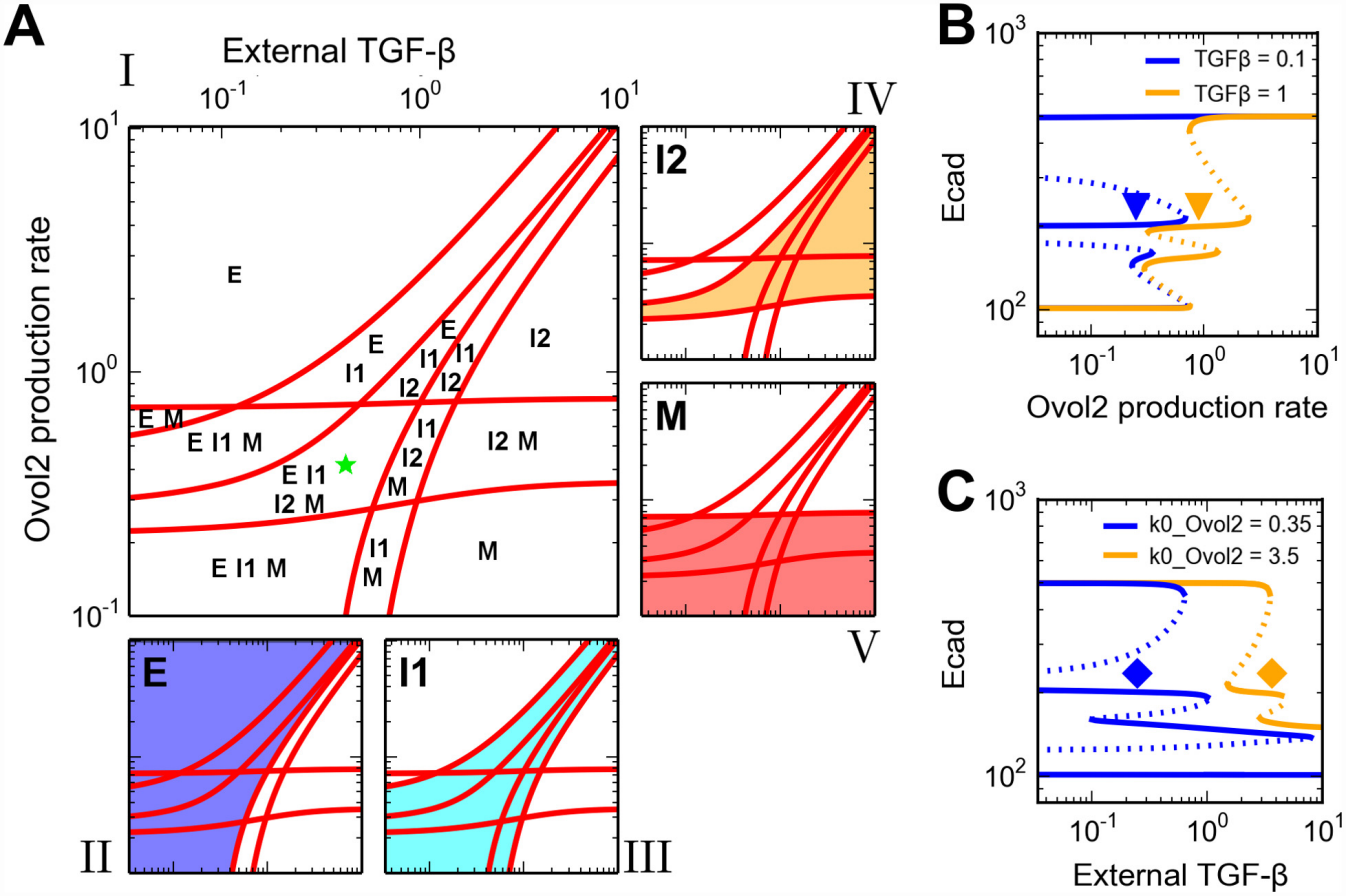
Roles of EMT-promoting and -inhibiting factors in the four-state EMT system. **A)** Two-parameter bifurcation diagram with respect to external TGF-β and Ovol2 basal production rate. The red curves were computed by extending the saddle-node bifurcation points obtained in one parameter bifurcation analysis (S4 Fig), and they define different parameter regions that can be mono-stable, bi-stable, tri-stable or tetra-stable depending on the number of possible stable phenotypes (see labels), and each multi-stable region can be viewed as an area where multiple phenotypes co-exist (II-V). The size of each phenotype region is an indication of robustness of the phenotype when the two signals are varied. Green star: a basal parameter set and an intermediate TGF-β concentration that together give rise to four phenotypes. **B, C)** One-parameter bifurcation diagrams of Ecad with respect to external TGF-β (B) and Ovol2 basal production rate (C). Solid curve: stable steady state. Dashed curve: unstable steady state. A basal parameter set (blue) and a perturbed parameter set (orange) are compared in each plot. Triangles and diamonds denote the conditions under which both I1 and I2 are stable.

First, we explored the roles of Ovol2 in regulatory control of the four states. Through bifurcation analysis with respect to external TGF-β and basal production rate of Ovol2 representing examples of EMT-inducing and -suppressing signals that are responsive to changes of the tissue microenvironment, we found these two signals to produce various combinations of cell phenotypes (Fig 6A(I)). Clearly, Ovol2 basal production rate exerted positive and negative effects on the robustness of E and M states, respectively (Fig 6A, blue and pink areas), and this is consistent with the demonstrated role of Ovol2 in preventing EMT and inducing MET ([11], [13] and this study). The effect of Ovol2 on the two intermediate states can be either positive or negative. Stability of I1 can be maintained when the strengths of Ovol2 and TGF-β signals are approximately proportional, with low levels of both giving rise to the most robust condition (Fig 6A, cyan area). In contrast, stability of I2 requires a minimum rate of Ovol2 basal production, but its robustness increases with higher levels of both Ovol2 and TGF-β (Fig 6A, orange area). In a specific case, when TGF-β signal was increased by 10-fold, higher Ovol2 basal production rate was required to retain the stability of both I1 and I2 states and to prevent the cells from differentiating into M state (Fig 6B, blue and orange triangles). Conversely, when Ovol2 basal production rate was increased, higher TGF-β signal strength was required to retain two stable intermediate states and to prevent the cells from differentiating into E state (Fig 6C, blue and orange diamonds). Overall, our analysis suggests that Ovol2 production tends to stabilize E state and destabilize M state, and that the two intermediate states are favored in two distinct conditions (high- versus low-signals) but both require the proper balance between EMT-inducing (e.g. TGF-β) and -suppressing signals (e.g. signals that induce Ovol2 expression).

**Fig 7:**
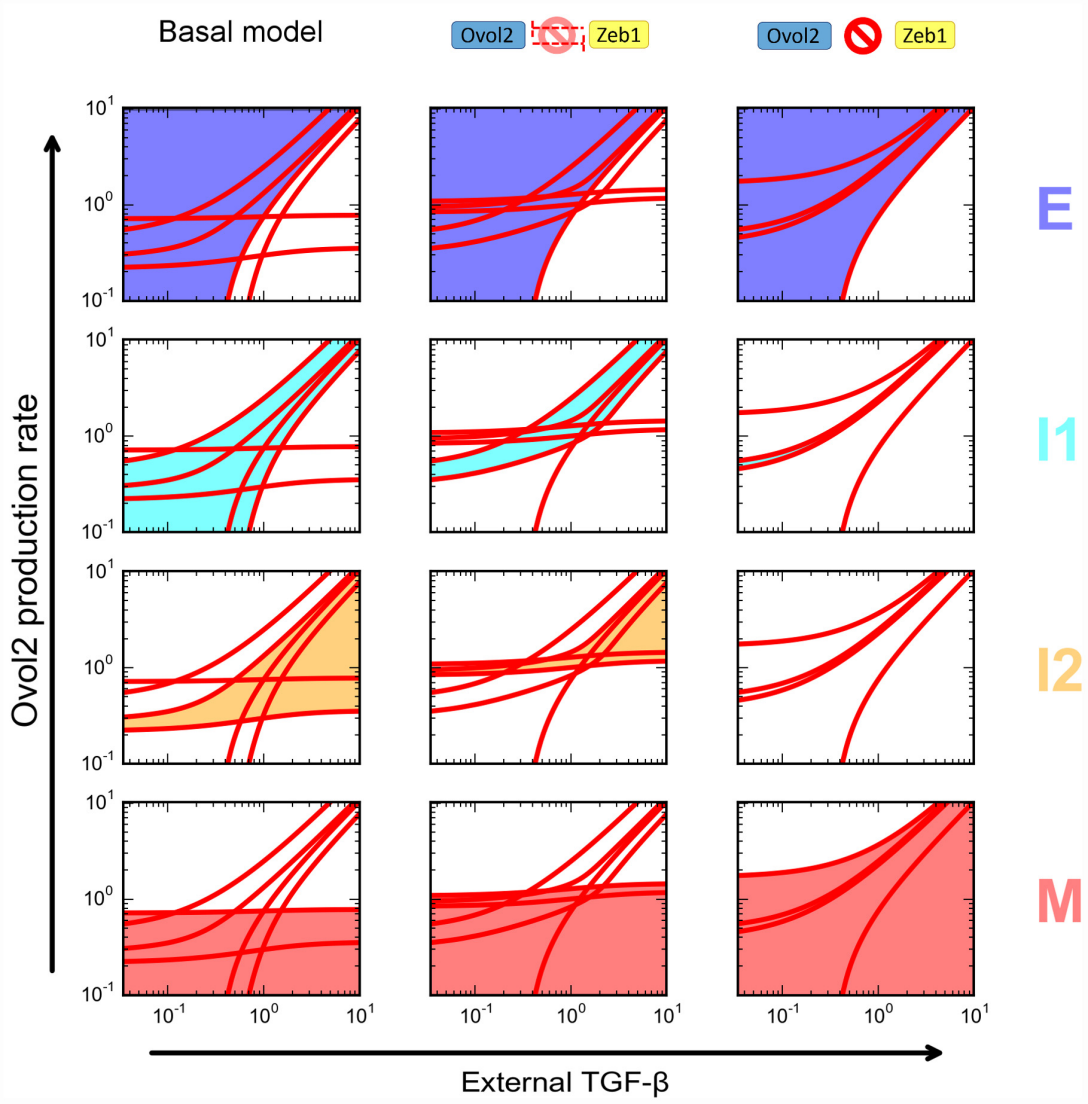
Roles of the Ovol2-Zeb1 mutual inhibition loop in the four-state EMT system. Comparison of the basal model (left column), reduced Ovol2-Zeb1 mutual inhibition (middle column), and blocked Ovol2-Zeb1 mutual inhibition (right column) on the four phenotypes. Each subplot is a two-parameter bifurcation diagram similar to Fig 6A. Subplots in each column highlight the various phenotypes in one condition. Shaded areas are highlighted phenotypes. Colors of the shading correspond to the colored labels on the right.

**Fig 8:**
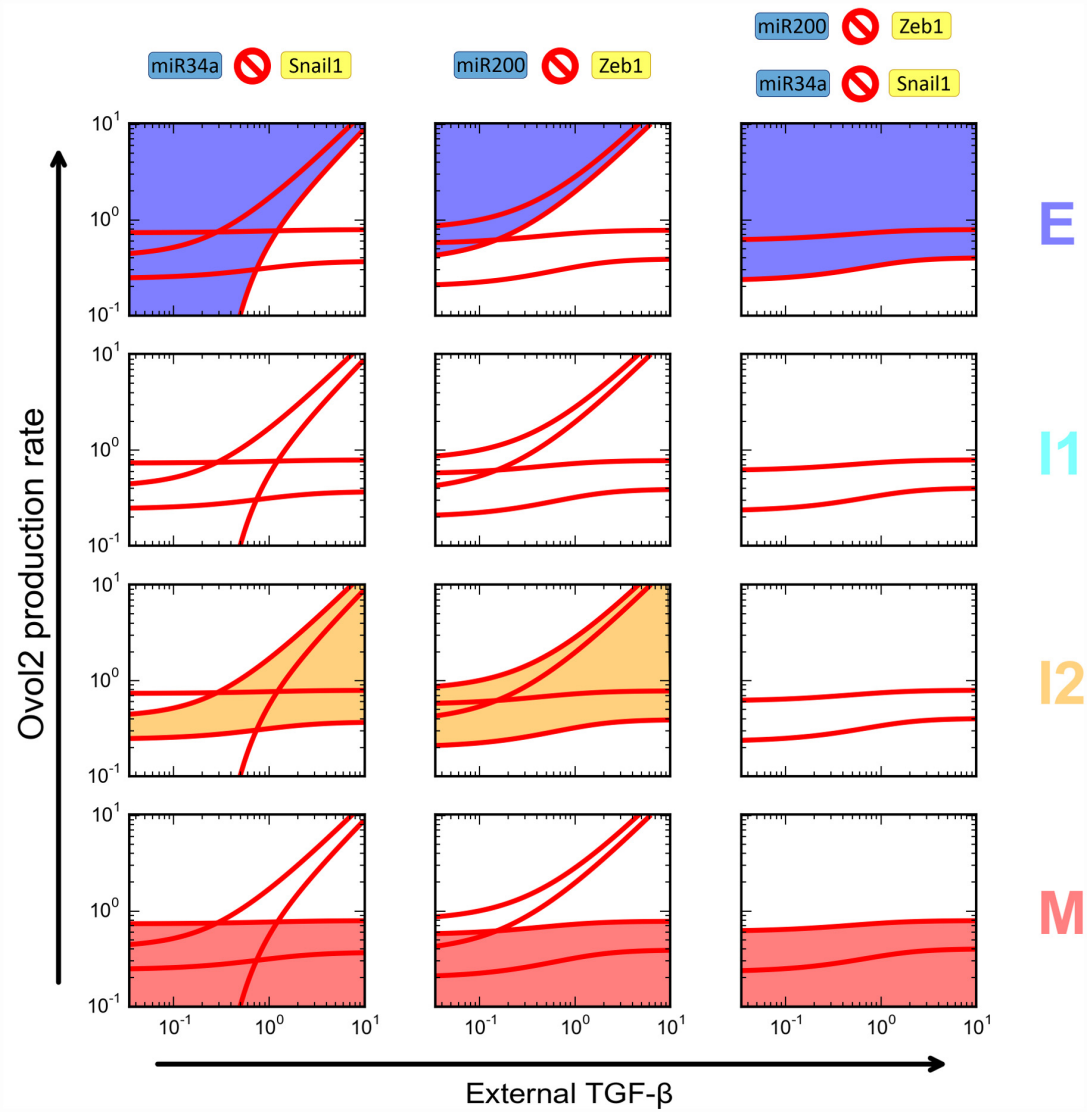
Roles of the miR34a-Snail and miR200-Zeb1 mutual inhibition loops in the four-state EMT system. Comparison of removing miR34a-Snail mutual inhibition (left column), miR200-Zeb1 mutual inhibition (middle column), or both (right column) on the four phenotypes. Each subplot is a two-parameter bifurcation diagram similar to Fig 6A. Subplots in each column highlight the various phenotypes in one condition. Shaded areas are highlighted phenotypes. Colors of the shading correspond to the colored labels on the right.

Next we reduced the strength of the Zeb1-Ovol2 mutual inhibition loop to determine its specific role in the four-state system. This led to no significant effect on the robustness of E state, moderately positive effect on that of M state (Fig 7, middle column, blue and pink areas), but significantly reduced robustness of the two intermediate states (Fig 7, middle column, cyan and orange areas). A complete blockage of the mutual inhibition loop resulted in a very small I1 region, and complete disappearance of the I2 region (Fig 7, right column). The role of the Ovol2-Zeb1 loop appeared distinct from that of the miR34a-Snail and miR200-Zeb1 mutual inhibition loops, as at least one of the miRNA-TF loops becomes dispensable for I2 (but not I1) when the other is intact (Fig 8, left and middle columns). This said, a complete blockage of both miRNA-TF loops abolished the intermediate states (Fig 8, right column). Partial blockage of each mutual inhibition loop gave rise to complex effects on the robustness of the two intermediate states (Table 1 and S5 Fig), but these effects are consistent with the finding of redundancy between the two miRNA-TF loops in terms of maintaining I2 state. Interestingly, partial blockage of miR200-Zeb1 resulted in a merge of the I1 and I2 regions, forming a large, continuous intermediate region (S5 Fig). Collectively, these results suggest that while all three mutual inhibition loops contribute to the existence and robustness of the two intermediate states, the strength of the Ovol2-Zeb1 loop is more critical.

**Table 1:** Influence of blocking mutual inhibition loops on the two intermediate states.

Mutual inhibition loop	Knockdown condition	Example of parameter setting	Major effect on intermediate states
miR34a-Snail	Partial removal	0.1% 1/*J*2_200_	Disappearance of I1 and decrease of I2
		25% *K*_*SR*_, 25% 1/*J*1_34_	Decrease of both I1 and I2
		50% *K*_*SR*_, 50% 1/*J*1_34_	Decrease of I1
		0.1% *K*_*SR*_	Disappearance of I1 and increase of I2
	Complete removal	0.1% *K*_*SR*_, 0.1% 1/*J*1_34_	
miR200-Zeb1	Partial removal	90% 1/*J*2_200_	Decrease of I2
		75% 1/*J*2_200_	Merge of I1 and I2
		50% *K*_1_	Decrease of I1 and increase of I2
		25% *K*_1_, 25% 1/*J*2_200_	Disappearance of I1 and increase of I2*
	Complete removal	0.1% *K*_1_, 0.1% 1/*J*2_200_	
miR34a-Snail and miR200-Zeb1	Complete removal	0.1% *K*_*SR*_, 0.1%1/*J*1_34_, 0.1% *K*_1_, 0.1% 1/*J*2_200_	Disappearance of both I1 and I2
Ovol2-Zeb1	Partial removal	50% 1/*J*_*O*_	Increase of I2
		50% 1/*J*2_*zeb*_	Decrease of I1 and decrease of I2
		25% 1/*J*_*O*_, 25% 1/*J*2_*zeb*_	Decrease of I1 and disappearance of I2
	Complete removal	0.1% 1/*J*_*O*_, 0.1% 1/*J*2_*zeb*_	Tiny I1 region and disappearance of I2

* This effect may also be interpreted as ‘I1 and I2 merged to form a large intermediate state’, but this intermediate state is closer to I2 than to I1 under this condition.

### Distinct differentiation propensities of the two intermediate states

**Fig 9:**
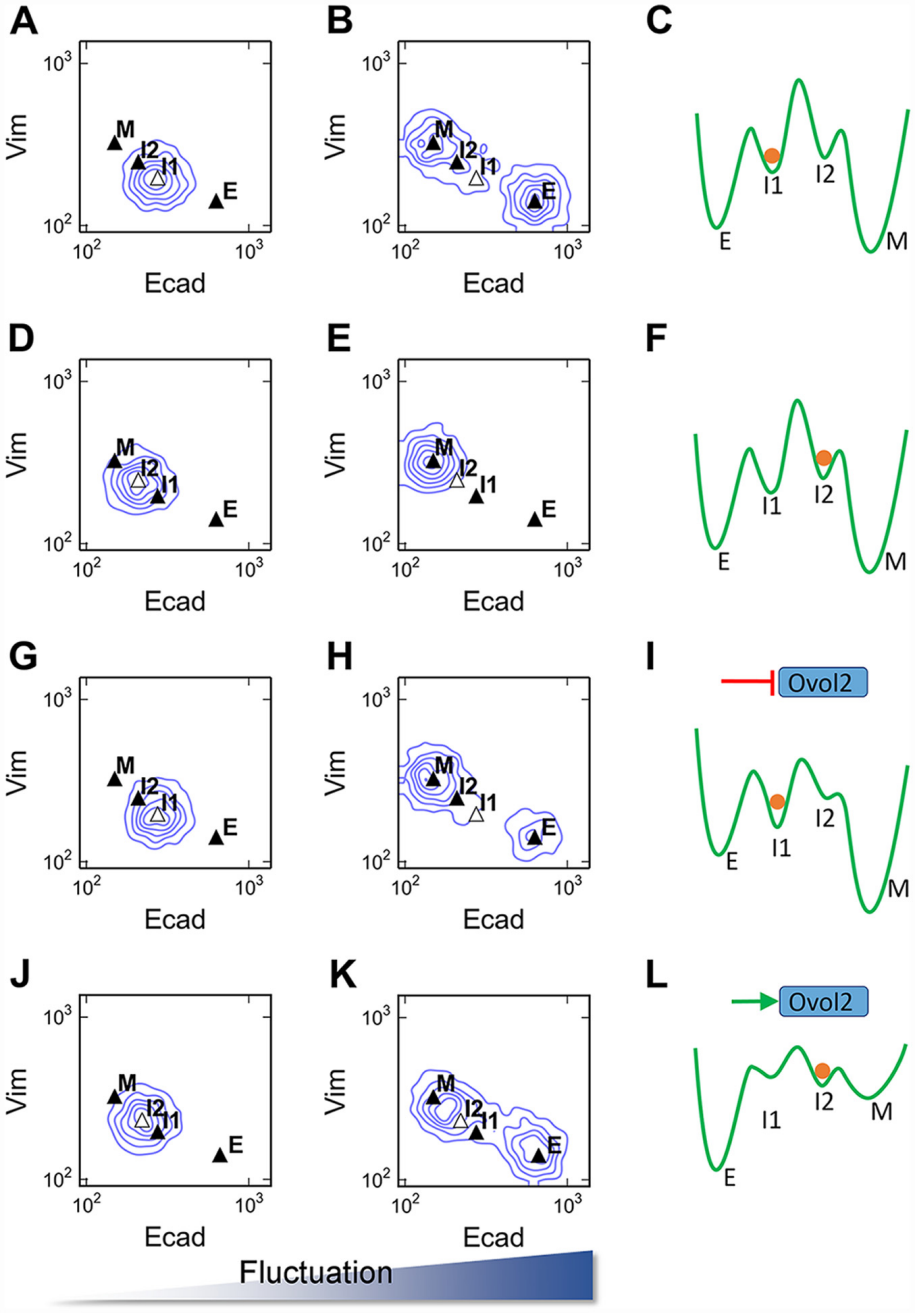
Distinct differentiation propensities of the two intermediate states. **A, B, D, E)** Stochastic simulations for a population of 5000 cells in four different conditions. Basal parameter set and intermediate external TGF-β concentration (0.5) were used (green star in Fig 6A). **A)** Initial condition: I1; small fluctuations. **B)** Initial condition: I1; large fluctuations. **D)** Initial condition: I2; small fluctuations. **E)** Initial condition: I2; large fluctuations. **G, H)** Stochastic simulations for a population of 5000 cells initially at I1 state. Ovol2 basal production level was reduced by 20% from basal parameter. **J, K)** Stochastic simulations for 5000 cells initially at I2 state. Ovol2 basal production level was increased by 100% from basal parameter. **C, F, I, L)** Metaphoric energy landscapes (green curves) for I1 **(C)**, I2 **(F)** initial conditions, and reduced **(I)** or increased **(L)** Ovol2 basal expression rate. Orange circle represents the initial condition.

We next used stochastic modeling to examine how likely a population of cells at the two intermediate states differentiates into E or M state when gene/protein expression fluctuates. We chose a condition under which the four states are stable (Fig 6A, green star). Simulations were performed under this condition for two populations of cells originating from I1 and I2 respectively (Fig 9A and 9F). When fluctuations were small, cells stayed in the basins of attraction of their initial steady state by the end of the simulation (Fig 9A and 9D). Large fluctuations triggered both E(I)MT and M(I)ET of the cells originally in I1, resulting in a heterogeneous population containing E and M phenotypes, whereas the same level of fluctuations triggered E(I)MT of the cells originally in I2 (Fig 9B and 9E and S6 Fig). Thus, the I1 and I2 cells have distinct differentiation propensities, with I1 cells more likely differentiating into E state, whereas I2 cells more likely into M state. These simulations also allowed us to infer that E and M states are more stable than the intermediate states, and that I1 is more stable than I2 under the particular conditions tested (Fig 9C and 9F).

We also asked whether changing Ovol2 production rate can affect the differentiation propensities from the I1 state. We reduced the basal production rate of Ovol2 by 20% and performed stochastic simulations starting from I1 as in Fig 9A and 9B. We found that this reduced Ovol2 production rate enabled more cells to settle at M state, and less cells to settle at I1 or E state (compare Fig 9B and 9H). We speculate that this is due to the reduced stability of E and I1 states, and/or the reduced energy barrier from I1 to I2 and M states (Fig 9I), providing a possible thermodynamic explanation for the role of Ovol2 in preventing EMT and inducing MET. Conversely, increased basal production of Ovol2 enabled some of the cells from I2 state to settle at E state instead of M state in the presence of large fluctuations (Fig 9J, 9K and 9L; compare panels E and K). These results suggest that the differentiation propensities of the two intermediate states can be regulated by tuning the level of Ovol2 expression.

## Discussion

Our study provides both modeling and experimental evidence for a new intermediate state that lies between E and M states in addition to the recently observed intermediate state [9,10,14]. Previous studies based on epigenetic modifications predicted that multiple intermediate states may exist between terminal E and M states, and they may contribute to phenotypic plasticity in a continuous manner [15]. Additionally, Huang et al. classified 43 ovarian carcinoma cell lines into four subgroups, including an E-like intermediate and an M-like intermediate states in the EMT spectrum, based on expression patterns of signature EMT genes [20]. To our knowledge, our work is the first unequivocal demonstration of two intermediate states in EMT. Previous theoretical study revealed four types of stable states during T cell differentiation [21]; a common feature of that and our study is the inclusion of multiple (a minimum of three) positive feedback loops (including mutual inhibition). We anticipate that as the complexity of modeling increases by adding more regulatory elements, even more intermediate states may be observed, with the most extreme scenario being a spectrum of metastable or stable cell phenotypes lying between the terminal E and M states. A unique and interesting feature of our four-state model is that intermediate states are not necessarily metastable; instead they can be stable with no (I1) or high (I2) EMT-inducing/inhibitory forces. It is the balance between these two opposing forces that is critical for maintaining the intermediate states.

Given the assumptions we make in our mathematical model, we have shown that the Ovol2-Zeb1 mutual inhibition loop is necessary for maintaining a four-state system. On one hand, this highlights the unique importance of the role of Ovol2 in EMT. On the other hand, the model leaves open the possibility that a four-state system could be governed by other unknown TFs that might be involved in a similar mutual inhibition loop. As discussed above, with additional positive feedback loops (>3), it is conceivable that additional intermediate states will emerge. As such, our model provides a framework for identifying and analyzing multiple intermediate phenotypes in EMT, and suggests a general and unique role of TF-based mutual inhibition loop in this system. With a proposed Ovol-Zeb1 mutual inhibition loop, a recent modeling study suggested important roles of Ovol2 in controlling the previously established three-state EMT system [22]. This is in agreement with our findings that Ovol2 is critical for both intermediate states.

What is the advantage of having intermediate state(s)? Such state can be a “hybrid” state, where cells exhibit both, albeit partial, “E” and “M” phenotypes. Indeed, during mammary gland morphogenesis, some epithelial cells at the tips of growing ducts express mesenchymal markers while simultaneously retaining epithelial integrity, suggesting that they are in a naturally occurring “hybrid” state [23]. In this case, a “hybrid” phenotype would enable the cells to undergo collective migration, by which they invade the surrounding stroma as a coherent epithelial front to facilitate branching morphogenesis. The same may be true for metastatic cancer cells as they acquire a mesenchymal phenotype to invade the surrounding tissue and colonize distant sites as epithelial tumors. Alternatively, an intermediate state can be a “naïve” state, where cells are devoid of typical epithelial and mesenchymal features. Along this line, we note our experimental observation that MCF10A cells seem to first lose their initial phenotypes (E or M), and then gain their destination phenotypes (M or E) during the factors-directed state transitions (Fig 3). Traveling through a “naïve” state could be a useful mechanism to erase memories of old lineages, thus creating a window of opportunity for expanded differentiation plasticity as desired for multipotent stem cells.

Why multiple intermediate states then? Chuong and Widelitz proposed the interesting idea that stem cell states can be regulated depending on the physiological needs of tissues to generate different numbers of intermediate stops on their journey to differentiation [24]. The same concept may be applicable to the EMT system, as having multiple intermediate states offers additional facets of regulation to more precisely control the temporal and spatial flux of epithelial cells through their differentiation/dedifferentiation pathway to adapt to various tissue environments or topology. Regardless of whether cells adopt an intermediate fate to gain "hybrid" behavior (e.g., collective migration) or to dedifferentiate to a naive state for lineage plasticity, the more intermediate states there are, the more thermodynamic traps that would be. Thus having more than one intermediate state would create a more controllable energy barrier so that cells do not easily fall into the mesenchymal state, which we know from previous studies is largely irreversible [14]. Non-genetic heterogeneity and spontaneous conversion among subpopulations have been documented for hematopoietic stem cells and breast cancer cells [4,25–27]. Theoretical analysis of these dynamic processes often involves the assumption that gene regulatory networks can generate multiple stem-like states that are adjacent in state space [25,26,28,29]. Our model presents a good example in which a network of three mutual inhibition loops is capable of giving rise to two adjacent states that may be associated with subpopulations of cells having distinct propensities for differentiation or tumorigenesis. The unstable nature of the I2 state under conditions examined and the phenotypic similarities between I1 and I2 states prevented us from further characterizing the molecular differences between the two intermediate cell populations and their corresponding cellular behaviors. As such, the functional significance of having two intermediate states has yet to be experimentally established.

We have demonstrated bidirectional transitions of MCF10A cells upon Zeb1/Snail overexpression (I1-M transition), TGF-β treatment [I1-(I2)-M transition] and Ovol2 overexpression (I1-E transition) (Fig 5B). It is tempting to ask which extracellular signaling molecules can trigger Ovol2 upregulation and the subsequent transition to E state under physiological conditions. Among the possible candidates is a BMP signal as it is known to induce MET [11,13] and to positively regulate Ovol2 expression during embryonic stem cell differentiation [30]. Identification of Ovol2-inducing external signals that can induce MET of MCF10A cells will enable a finer analysis of the dynamic process of MET as well as further experimental validation of our mathematic model.

In summary, our work identifies transcriptional factor Ovol2 and its mutual inhibition relationship with Zeb1 as critical additions to the known EMT regulatory network. Specifically, these new regulatory elements are important for attaining and maintaining the two intermediate states. Furthermore, their experimental perturbations allowed us to observe the bidirectionality of transitions from the intermediate states. Together, our study offers a framework for understanding the molecular strategies and design principles by which epithelial stem, progenitor, or cancer cells achieve multipotency or collective migration.

## Methods

### Cell lines

MCF10A, MCF7, MDA-MB-231 were purchased from ATCC. MCF7 and MDA-MB231 cells were maintained in Dulbecco’s modified Eagle’s medium (DMEM) supplemented with 10% fetal bovine serum. MCF10A cells were grown in DMEM/F12 (1:1) medium with 5% horse serum, epidermal growth factor (10 ng/mL), cholera toxin (100 ng/mL) and insulin (0.023 IU/mL). For TGF-β treatment, cells were incubated with titrated concentrations of human TGF-β1 protein (R&D systems) in complete culture medium for 10 days. The culture medium was replaced daily and cells were passaged just before reaching full confluency.

### Lentiviral expression system

Recombinant lentiviruses expressing Ovol2 using the pHIV-ZsGreen lentiviral construct was described previously [11]. For Snail and Zeb1 expression, human SNAIL cDNA and mouse Zeb1 cDNA were cloned into the XhoI/NotI and EcoRI sites of pHIV-ZsGreen, respectively. Production and infection of lentiviruses were carried out as previously described [11]. Transduction unit of viral solution was estimated by measuring GFP-positive population using a flow cytometer.

### Flow cytometry

For Ecad/Vim profiling, cells were fixed with 4% paraformaldehyde, permeabilized with 0.2% Triton X-100, and stained with the following primary and secondary antibodies and reagents: anti-E-cadherin (Life Technologies, 1:500), anti-vimentin (Cell Signaling Technologies, 1:500), allophycocyanin (APC)-labeled anti-mouse IgG (Santa Cruz), Cy3-labeled anti-rabbit IgG (Jackson Immuno). For CD44 staining, live cells were stained with phycoerythrin (PE)-conjugated anti-CD44 antibody (Biolegend). Live-cell sorting for GFP^+^ cells was performed on a BD FACSAria equipped with FACS DiVa6.0 software operating at low pressure (20 psi) using a 100-μm nozzle. Cell clusters and doublets were electronically gated out. Cells were routinely double sorted and post-sort analysis typically indicated purities of >90% with minimal cell death (<10%).

### ChIP assay

ChIP was performed with an anti-Zeb1 antibody (Santa Cruz) according to the previously described protocol [11]. The following primers were used to detect Ovol2 promoter regions: proximal site (forward, 5’-GTGATAGGGGTATGAAGCAGAGG-3’ reverse, 5’-CACCAGGAAACTTGGGAGTG-3’) and distal site (forward, 5’-AGCCCAGAAATCCGTTACCA-3’ reverse, 5’-CTCACTGCTGGAGGTTGTCT-3’).

### RT-PCR

Total RNA was isolated using the TRIzol Reagent (Invitrogen) followed by cleaning up and RNase-free DNaseI treatment using the RNeasy mini kit (QIAGEN). cDNA was prepared using Retroscript Kit (Applied Biosystems) according to manufacturer’s instructions. Real-time PCR was performed using a CFX96 qPCR system and SsoAdvanced SYBR Green Supermix (Bio-rad). Comparative analysis was performed between the genes of interest normalized by the house keeping genes *GAPDH* and *ACTB*. The following primers were used: *OVOL2* (forward, 5’-AGCTGTGACCTGTGTGGCAAG-3’ reverse, 5’-ACGAATGCCTGTGTGTGTGC-3’), *ZEB1* (forward 5’-TTGCTCCCTGTGCAGTTACA-3’ reverse 5’-CGTTTCTTGCAGTTTGGGCA-3’), *GAPDH* (forward, 5’-GGACCTGACCTGCCGTCTAGAA-3’ reverse, 5’-GGTGTCGCTGTTGAAGTCAGAG-3’), and *ACTB* (forward, 5’-CTTCTACAATGAGCTGCGTG-3’ reverse, 5’-GGGTGTTGAAGGTCTCAAAC-3’). For semi-quantitative PCR, the following primers were used: *CDH1* (forward, 5’-AAAGGCCCATTTCCTAAAAACCT-3’ reverse, 5’-TGCGTTCTCTATCCAGAGGCT-3’), *SNAI1* (forward, 5’-CCTCCCTGTCAGATGAGGAC-3’ reverse, 5’-CCAGGCTGAGGTATTCCTTG-3’), *VIM* (forward, 5’-GACGCCATCAACACCGAGTT-3’ reverse, 5’-CTTTGTCGTTGGTTAGCTGGT-3’).

### Mathematical modeling

We used ordinary differential equations (ODEs) to model the regulatory network shown in Fig 2A. The framework of the model stems from a recently published EMT model [14], and the modeling details are described therein. This framework employs mass-action dynamics to model microRNA-mRNA interactions with considerations of the microRNA binding sites on their targets. This modeling strategy was introduced by Lu et al. [9,31]. As other transcription factor regulations, interactions involving Ovol2 were modeled with Hill functions. Numerical bifurcation analysis was performed with PyDSTool [32]. To consider fluctuations in gene expression, we added multiplicative white noise to some of the ODEs. To determine which phenotype (basin of attraction) a cell adopts at the end of the simulations, we set the noise terms to zero and let the simulation continue until it reached steady state (S6 Fig). Lists of equations, parameters and assumptions can be found in supplementary materials. Stochastic simulations were performed with XPPAUT [33].

## Supporting Information

S1 Text Details of mathematical model. (DOCX)

S1 Table Experimental evidence supporting influence diagram. (DOCX)

S2 Table List of basal parameter values. (DOCX)

S1 Fig MCF10A cells show intermediate expression levels of EMT-related genes. RT-semi-quantitative PCR analysis of the indicated genes in three human breast cell lines. Two biological replicates were performed for each gene in each type of cell line.
(TIF)

S2 Fig FACS profiles of epithelial/mesenchymal phenotypes upon forced expression of the indicated TFs. Successfully infected population (GFP-positive) can be distinguished from the uninfected population (GFP-negative) by GFP fluorescence (left panels). Ecad/Vim profiles are visualized separately for GFP-positive and–negative populations. Analysis was performed on MCF10A cells five days after infection. Note that GFP-negative population serves as an internal control. Only GFP-positive populations were analyzed in the experiments for main figures.
(TIF)

S3 Fig Stochastic simulations for stepwise I1-I2-M transitions upon TGF-β treatment. Stochastic simulation (started at I1 state) for a population of 2000 cells at three concentration of TGF-β. Green: no TGF-β (I1 state). Red: high (10 units) TGF-β concentration (M state). Cyan: intermediate (2.5 units) TGF-β concentration (a mixture of I2 and M populations can be obtained at the low-noise condition). At high TGF-β concentration, the system is monostable at M state. At intermediate TGF-β concentration, the system is bistable at M or I2 state. See Fig 2B.
(TIF)

S4 Fig Bifurcation diagram for Ecad with respect to external TGF-β. Solid curve: stable steady state. Dashed curve: unstable steady state. Red dots: saddle-node bifurcation points used for computing curves in Fig 6A.
(TIF)

S5 Fig Effects of partial blockage of mutual inhibition loops. Shown are comparisons of the basal model (leftmost column), complete blockage (rightmost column), and partial blockage (middle columns) of miR34a-Snail (A), miR200-Zeb1 (B) and Ovol2-Zeb1 (C) mutual inhibition loops on the four phenotypes. Each subplot is a two-parameter bifurcation diagram similar to Fig 6A. Subplots in each column highlight the various phenotypes in one condition. Shaded areas are highlighted phenotypes. Colors of the shading correspond to the colored labels on the right.
(TIF)

S6 Fig Stochastic transitions from I1 state to E and M states. Stochastic simulations for a population of 2000 cells. The basal parameter set and initial condition at I1 used (as in Fig 9). TGF-β concentration was raised from 0 to 0.5 at t = 100. White noise terms were set to zero at t = 500.
(TIF)
