# Supplementary material for: An Ovol2-Zeb1 Mutual Inhibitory Circuit Governs Bidirectional and Multi-step Transition between Epithelial and Mesenchymal States
Source: PLoS Comput Biol. 2015 Nov 10;11(11):e1004569. doi: 10.1371/journal.pcbi.1004569 (PMC4640575; doi:10.1371/journal.pcbi.1004569)
Supplement: S1 Text — (DOCX) [file pcbi.1004569.s001.docx]

**An Ovol2-Zeb1 Mutual Inhibitory Circuit Governs Bidirectional and Multi-step Transition between Epithelial and Mesenchymal States**

Tian Hong^1, 2¶^, Kazuhide Watanabe^2, 3¶^, Catherine Ha Ta^1, 2^, Alvaro Villarreal-Ponce^2, 3^, Qing Nie^1, 2*^ and Xing Dai^2, 3*^

^1^ Department of Mathematics,

^2^ Center for Complex Biological Systems

^3^ Department of Biological Chemistry, School of Medicine,

University of California, Irvine, CA 92697, USA

¶ These authors contributed equally.

* Corresponding authors.

**Supporting Text S1**

**Details of mathematical model**

**1. Model construction.**

**2. Stochastic simulation.**

**3. Analysis of the effects of specific regulations on cell phenotypes.**

**1. Model construction**

The framework of our model is from a recent publication by Zhang et al. [[1](#_ENREF_1)]. In that study, Zhang et al. demonstrated that both Zeb1-miR-200 and Snail1-miR-34 loops can create bistable switches. Therefore, this published model is also referred to as Cascading Bistable Switches (CBS) model in that paper and herein. We added the following interactions to the existing network in CBS model in order to consider regulations involving Ovol2:

1. Ovol2 inhibits Zeb1 expression [[2](#_ENREF_2),[3](#_ENREF_3)]

2. Ovol2 inhibits TGF-β expression [[2](#_ENREF_2)]

3. Ovol2 inhibits TGF-β signaling, partly by inhibiting Snail expression [[2](#_ENREF_2),[4](#_ENREF_4)]

4. Zeb1 inhibits Ovol2 expression (evidence from this study)

Other regulations and the supporting experimental evidence are listed in Table S1.

Details of modeling framework can be found in [[1](#_ENREF_1)]. Briefly, law of mass action was used to describe miRNA-mRNA interaction, and miRNA-mediated mRNA degradation was explicitly considered. This framework was first introduced by Lu et al. [[5](#_ENREF_5),[6](#_ENREF_6)]. Generalized Hill function was used to describe transcriptional activation and inhibition by transcription regulators other than miRNAs. We adjusted some parameter values from the CBS model in order to examine the qualitative dynamic behavior that might be introduced by Ovol2-Zeb1 interactions.

Our model has 18 ordinary differential equations (ODEs) as follows:

$$\begin{matrix} \frac{\text{d[}snail1\text{]}_{\text{t}}}{\text{d}t}\text{=}k0_{snail}+k_{snail}\frac{(([TGF]+[TGF0])/J0_{snail})^{n0_{snail}}}{(1+(([TGF]+[TGF0])/J0_{snail})^{n0_{snail}}+([OVOL2]/J1_{snail})^{n1_{snail}})(1+[SNAIL]/J2_{snail})} \\ -kd_{snail}([snail1\text{]}_{\text{t}}-\text{[}SR])\text{ }-kd_{SR}\text{[}SR\text{]} \end{matrix}$$

$$\frac{\text{d[}SNAIL\text{]}}{\text{d}t}\text{=}k_{SNAIL}\text{([}snail1\text{]}_{\text{t}}-\text{[}SR])\text{ }-\text{ }kd_{SNAIL}[SNAIL]$$

$$\frac{\text{d[}miR34\text{]}_{\text{t}}}{\text{d}t}\text{=}k0_{34}\text{+}\frac{k_{34}}{\text{1+([}SNAIL]/J1_{34}\text{)}^{n1_{34}}\text{+([}ZEB]/J2_{34}\text{)}^{n2_{34}}}-kd_{34}\text{([}miR34\text{]}_{\text{t}}-[SR\text{])}-(1-\lambda_{SR})kd_{SR}\text{[}SR\text{]}$$

$$\frac{\text{d[}SR\text{]}}{\text{d}t}\text{=}Tk\text{(}K_{SR}\text{([}snail1\text{]}_{\text{t}}-\text{[}SR\text{])([}miR34\text{]}_{\text{t}}-\text{[}SR])-[SR\text{])}$$

$$\begin{matrix} \frac{\text{d[}zeb\text{]}_{\text{t}}}{\text{d}t}\text{=}k0_{zeb}+k_{zeb}\frac{([SNAIL]/J1_{zeb})^{n1_{zeb}}}{1+([SNAIL]/J1_{zeb})^{n1_{zeb}}+([OVOL2]/J2_{zeb})^{n2_{zeb}}}-kd_{zeb}([zeb\text{]}_{\text{t}}-\sum_{i=1}^{5} \text{C}_{5}^{i}\text{[}ZR_{i}\text{])} \\ -\sum_{i=1}^{5} kd_{ZR_{i}}\text{C}_{5}^{i}\text{[}ZR_{i}\text{]} \end{matrix}$$

$$\frac{\text{d[}ZEB\text{]}}{\text{d}t}\text{=}k_{ZEB}([zeb\text{]}_{\text{t}}-\sum_{i=1}^{5} \text{C}_{5}^{i}\text{[}ZR_{i}\text{])}-kd_{ZEB}[ZEB\text{]}$$

$$\begin{matrix} \frac{\text{d[}miR200\text{]}_{\text{t}}}{\text{d}t}\text{=}k0_{200}+k_{200}\frac{1}{1+([SNAIL]/J1_{200})^{n1_{200}}+([ZEB]/J2_{200})^{n2_{200}}}-kd_{200}([miR200\text{]}_{\text{t}}-\sum_{i=1}^{5} i\text{C}_{5}^{i}\text{[}ZR_{i}\text{]} \\ -\text{[}TR\text{])}-\sum_{i=1}^{5} (1-\lambda_{i})kd_{ZR_{i}}\text{C}_{5}^{i}i\text{[}ZR_{i}\text{]}-(1-\lambda_{TR})kd_{TR}[TR] \end{matrix}$$

$$\frac{\text{d[}ZR_{1}\text{]}}{\text{d}t}\text{=}Tk(K_{1}([miR200\text{]}_{\text{t}}-\sum_{i=1}^{5} i\text{C}_{5}^{i}[ZR_{i}]-[TR])([zeb\text{]}_{\text{t}}-\sum_{i=1}^{5} \text{C}_{5}^{i}[ZR_{i}])-\text{[}ZR_{1}\text{])}$$

$$\frac{\text{d[}ZR_{2}\text{]}}{\text{d}t}\text{=}Tk(K_{2}([miR200\text{]}_{\text{t}}-\sum_{i=1}^{5} i\text{C}_{5}^{i}[ZR_{i}]-[TR])[ZR_{1}]-[ZR_{2}\text{])}$$

$$\frac{\text{d[}ZR_{3}\text{]}}{\text{d}t}\text{=}Tk(K_{3}([miR200\text{]}_{\text{t}}-\sum_{i=1}^{5} i\text{C}_{5}^{i}[ZR_{i}]-[TR])[ZR_{2}]-[ZR_{3}])$$

$$\frac{\text{d[}ZR_{4}\text{]}}{\text{d}t}\text{=}Tk(K_{4}([miR200\text{]}_{\text{t}}-\sum_{i=1}^{5} i\text{C}_{5}^{i}[ZR_{i}]-[TR])[ZR_{3}]-[ZR_{4}])$$

$$\frac{\text{d[}ZR_{5}\text{]}}{\text{d}t}\text{=}Tk(K_{5}([miR200\text{]}_{\text{t}}-\sum_{i=1}^{5} i\text{C}_{5}^{i}[ZR_{i}]-[TR])[ZR_{4}]-[ZR_{5}])$$

$$\frac{\text{d[}tgf\text{]}_{\text{t}}}{\text{d}t}\text{=}k_{tgf}-kd_{tgf}([tgf\text{]}_{\text{t}}-\text{[}TR])-kd_{TR}[TR\text{]}$$

$$\frac{\text{d[}TGF\text{]}}{\text{d}t}=k0_{TGF}+k_{TGF}([tgf\text{]}_{\text{t}}-[TR])-kd_{TGF}[TGF\text{]}$$

$$\frac{\text{d[}TR\text{]}}{\text{d}t}\text{=}Tk(K_{TR}([miR200\text{]}_{\text{t}}-\sum_{i=1}^{5} i\text{C}_{5}^{i}[ZR_{i}]-[TR])([tgf\text{]}_{\text{t}}-[TR])-[TR\text{])}$$

$$\frac{\text{d[}Ecad]}{\text{d}t}\text{=}k0_{E}+k_{E1}\frac{1}{1+([SNAIL]/J1_{E})^{n1_{E}}}+k_{E2}\frac{1}{1+([ZEB]/J2_{E})^{n2_{E}}}-kd_{E}[Ecad]$$

$$\frac{\text{d[}Vim\text{]}}{\text{d}t}\text{=}k0_{V}+(k_{V1}\frac{([SNAIL]/J1_{V})^{n1_{V}}}{1+([SNAIL]/J1_{V})^{n1_{V}}}+k_{V2}\frac{([ZEB]/J2_{V})^{n2_{V}}}{1+([ZEB]/J2_{V})^{n1_{V}}})/(1+[OVOL2]/J3_{V})-kd_{V}[Vim]$$

$$\frac{\text{d[}OVOL2\text{]}}{\text{d}t}\text{=}k0_{O}+k_{O}\frac{1}{1+([ZEB]/J_{O})^{n_{O}}}-kd_{O}[OVOL2]$$

where $\sum_{i=1}^{5} i\text{C}_{5}^{i}[ZR_{i}]=5[ZR_{1}]+2\times10[ZR_{2}]+3\times10[ZR_{3}]+4\times5[ZR_{4}]+5[ZR_{5}]$, which represents the total amount of miR-200 bound to *Zeb1* mRNA. Each term of this summation describes $\text{C}_{5}^{i}$ scenarios in which *i* number of miRNA molecules bind to $\text{C}_{5}^{i}$ possible binding sites that each *Zeb1* mRNA might have.

and $\sum_{i=1}^{5} \text{C}_{5}^{i}[ZR_{i}]=5[ZR_{1}]+10[ZR_{2}]+10[ZR_{3}]+5[ZR_{4}]+[ZR_{5}]$, which represents the total amount of complex formed by miR-200 and *Zeb1* mRNA.

Meanings of the state variables are as follows:

$\text{[}snail1\text{]}_{\text{t}}$ Concentration of *Snail1* mRNA

$\text{[}SNAIL\text{]}$ Concentration of Snail protein

$\text{[}miR34\text{]}_{\text{t}}$ Concentration of miR-34

$\text{[}SR\text{]}$ Concentration of complex formed by miR-34 and *Snai1l* mRNA

$\text{[}zeb\text{]}_{\text{t}}$ Concentration of *Zeb1* mRNA

$\text{[}ZEB\text{]}$ Concentration of Zeb1 protein

$\text{[}miR200\text{]}_{\text{t}}$ Concentration of miR-200

$\text{[}ZR_{i}\text{]}$ Concentrations of complexes formed by *i* miR-200 molecules and one *Zeb1* mRNA molecule

$\text{[}tgf\text{]}_{\text{t}}$ Concentration of *TGF-β* mRNA

$\text{[}TGF\text{]}$ Concentration of endogenous TGF-β protein

$\text{[}TR\text{]}$ Concentration of complex formed by miR-34 and *TGF-β* mRNA

$\text{[}Ecad]$ Concentration of Ecad protein

$\text{[}Vim\text{]}$ Concentration of Vim protein

$\text{[}OVOL2\text{]}$ Concentration of Ovol2 protein

**2. Stochastic simulations**

To consider the effect of molecular fluctuations in gene regulatory networks, we introduced uncorrelated multiplicative white noise to some of the ODEs as $\text{d}x/\text{d}t=f(x_{1},x_{2},...,x_{n})+\mu_{x}\cdot x\cdot\text{d}W$ , where $\text{d}W$is a Wiener process that can be discretized as $\text{d}W\sim N(0,1)/\sqrt{\text{d}t}$where $N(0,1)$ denotes a normally distributed random variable with 0 mean and unit variance, and $\mu_{x}$ represents the amplitude of the fluctuation. We noticed that the fluctuations on EMT -inducing and -repressing factors may have different effects on noise-induced phenotypic transitions (not shown), so we introduced the white noise to ODEs for Snail and Ovol2 to represent the noise on these two groups of factors. To adapt to the heterogeneous populations that we observed in flow cytometry experiments and to consider the additional fluctuations due to detection inaccuracy, we also introduced white noise to ODEs for Ecad and Vim. The basal values of the amplitude parameters for these four stochastic ODEs are $\mu_{Snail1}=0.02$, $\mu_{O}=0.2$**,** $\mu_{E}=0.02$ and $\mu_{V}=0.02$. To compare the effects of different levels of fluctuations, we increased $\mu_{SNAIL}$ and/or $\mu_{O}$ by 10-fold in some of our analysis (Fig. 3, S2 and S4). Larger fluctuations for Ecad and Vim ($\mu_{E}=0.07$ and $\mu_{V}=0.07$) were used to adapt to the experimental observations from flow cytometry (Fig. 3).

To unambiguously determine the phenotypes by the end of the stochastic simulations (*t*=500), we set the amplitude parameters $\mu_{x}$ to 0 at *t*=500 and continued the simulations for another 100 time units so that each trajectory is settled at a deterministic steady state (Fig. S4).

The high complexity of the system prevents us from more formal implementation of stochastic simulations such as Gillespie algorithms. A more systematic study of the effects of fluctuations and stochastic transitions among multiple states will be a subject of future studies.

**3. Analysis of the effects of specific regulations on cell phenotypes**

One-parameter bifurcation analysis was used to examine the change of the stable cell phenotypes in response to change of a particular parameter (e.g. external TGF-β concentration or basal production rate of Ovol2). Two-parameter bifurcation analysis was used to examine the phenotypic changes in response to both EMT -inducing and -repressing parameters/signals. The curves on the two-parameter bifurcation diagrams are continuation of the saddle-node bifurcation points that we obtained from the one-parameter bifurcation analysis (Fig. S3). To simulate the overexpression experiment, we increased in basal production rates of Ovol2 or Zeb1 by 10-fold (Fig. 3 and S2). To understand how each regulation of the network contributes to overall performance of the system, we used ‘knockdown’ approach in which we partially or completely removed certain regulations in the network and examined the effect on the two-parameter bifurcation diagrams (see Fig. 7). To simulate partial removal of Zeb1-Ovol2 mutual inhibition, we increased the Michaelis constants of the inhibitions between these two molecules ($J_{O}$ and $J2_{zeb}$) by 2-fold. For complete removal, we increased these parameters by 100-fold. Similar approaches were used to simulate complete removal of TF-microRNA mutual inhibition loops, i.e. $J1_{34}$ and/or $J2_{200}$ were increased by 100-fold, whereas$K_{1}$ and $K_{SR}$ **(**TF-microRNA binding/unbinding rate ratios**)** were decreased by 100-fold. Although it is challenging to systematically demonstrate the global effect of a particular regulation in a large parameter space, we assume that this approach gives a reasonable representation on their general functions. We did not analyze functions of isolated ‘modules’ in the network since they have been discussed extensively in previous computational analysis of the EMT system [[1](#_ENREF_1),[5-7](#_ENREF_5)]. Also, combination of mutual-inhibition and auto-activation loops is a well-studied dynamic system in general, so detailed analysis on Zeb1-Ovol2 module is not necessary.

**References**

1. Zhang J, Tian XJ, Zhang H, Teng Y, Li R, et al. (2014) TGF-β -induced epithelial-to-mesenchymal transition proceeds through stepwise activation of multiple feedback loops. Science Signaling 7: ra91-ra91.

2. Watanabe K, Villarreal-Ponce A, Sun P, Salmans ML, Fallahi M, et al. (2014) Mammary morphogenesis and regeneration require the inhibition of EMT at terminal end buds by Ovol2 transcriptional repressor. Developmental cell 29: 59-74.

3. Lee B, Villarreal-ponce A, Fallahi M, Ovadia J, Sun P, et al. (2014) Transcriptional Mechanisms Link Epithelial Plasticity to Adhesion and Differentiation of Epidermal Progenitor Cells. Developmental Cell 29: 47-58.

4. Peinado H, Quintanilla M, Cano A (2003) Transforming growth factor beta-1 induces snail transcription factor in epithelial cell lines: mechanisms for epithelial mesenchymal transitions. The Journal of biological chemistry 278: 21113-21123.

5. Lu M, Jolly MK, Levine H, Onuchic JN, Ben-Jacob E (2013) MicroRNA-based regulation of epithelial-hybrid-mesenchymal fate determination. Proceedings of the National Academy of Sciences of the United States of America 10.1073/pnas.1318192110.

6. Lu M, Jolly MK, Gomoto R, Huang B, Onuchic J, et al. (2013) Tristability in cancer-associated microRNA-TF chimera toggle switch. The journal of physical chemistry B 117: 13164-13174.

7. Tian X-J, Zhang H, Xing J (2013) Coupled reversible and irreversible bistable switches underlying TGFβ-induced epithelial to mesenchymal transition. Biophysical journal 105: 1079-1089.
