# Supplementary material for: An Ovol2-Zeb1 Mutual Inhibitory Circuit Governs Bidirectional and Multi-step Transition between Epithelial and Mesenchymal States
Source: PLoS Comput Biol. 2015 Nov 10;11(11):e1004569. doi: 10.1371/journal.pcbi.1004569 (PMC4640575; doi:10.1371/journal.pcbi.1004569)
Supplement: S1 Table — (DOCX) [file pcbi.1004569.s002.docx]

**Table S1. Experimental evidence supporting influence diagram**

| Molecular influence | Evidence |
| --- | --- |
| TGF-β upregulates *Snai1* transcription | [[1](#_ENREF_1)] |
| Snail upregulates *Zeb1* transcription | [[2](#_ENREF_2)] |
| Snail downregulates miR-34 transcription | [[3](#_ENREF_3),[4](#_ENREF_4)] |
| miR-34 downregulates Snail production | [[3](#_ENREF_3),[4](#_ENREF_4)] |
| Snail downregulates miR-200 transcription | [[5](#_ENREF_5)] |
| Snail downregulates its own transcription | [[6](#_ENREF_6)] |
| Snail downregulates Ecad production | [[7](#_ENREF_7),[8](#_ENREF_8)] |
| Snail upregulates Vim production | [[8](#_ENREF_8)] |
| Zeb1 downregulates miR-200 transcription | [[9](#_ENREF_9),[10](#_ENREF_10)] |
| miR-200 downregulates Zeb1 production | [[9](#_ENREF_9),[10](#_ENREF_10)] |
| Zeb1 downregulates miR-34 transcription | [[11](#_ENREF_11)] |
| Zeb1 downregulates Ecad production | [[12](#_ENREF_12)] |
| Zeb1 upregulates Vim production | [[13](#_ENREF_13)] |
| miR-200 downregulates TGF-β production | [[14](#_ENREF_14),[15](#_ENREF_15)] |
| Ovol2 downregulates *TGF-β3* transcription | [[16](#_ENREF_16)] |
| Ovol2 downregulates TGF-β signaling | [[16](#_ENREF_16)] |
| Ovol2 downregulates *Zeb1* transcription | [[16](#_ENREF_16),[17](#_ENREF_17)] |
| Zeb1 downregulates Ovol2 production | [[18](#_ENREF_18)]; this study |
| Ovol2 downregulates Vim production | [[16](#_ENREF_16),[17](#_ENREF_17)] |

**References**

1. Peinado H, Quintanilla M, Cano A (2003) Transforming growth factor beta-1 induces snail transcription factor in epithelial cell lines: mechanisms for epithelial mesenchymal transitions. The Journal of biological chemistry 278: 21113-21123.

2. Guaita S, Puig I, Franci C, Garrido M, Dominguez D, et al. (2002) Snail induction of epithelial to mesenchymal transition in tumor cells is accompanied by MUC1 repression and ZEB1 expression. The Journal of biological chemistry 277: 39209-39216.

3. Kim NH, Kim HS, Li X-Y, Lee I, Choi H-S, et al. (2011) A p53/miRNA-34 axis regulates Snail1-dependent cancer cell epithelial-mesenchymal transition. The Journal of cell biology 195: 417-433.

4. Siemens H, Jackstadt R, Hünten S, Kaller M, Menssen A, et al. (2011) miR-34 and SNAIL form a double-negative feedback loop to regulate epithelial-mesenchymal transitions. Cell cycle 10: 4256-4271.

5. Diaz-Lopez A, Diaz-Martin J, Moreno-Bueno G, Cuevas EP, Santos V, et al. (2015) Zeb1 and Snail1 engage miR-200f transcriptional and epigenetic regulation during EMT. Int J Cancer 136: E62-73.

6. Peiró S, Escrivà M, Puig I, Barberà MJ, Dave N, et al. (2006) Snail1 transcriptional repressor binds to its own promoter and controls its expression. Nucleic acids research 34: 2077-2084.

7. Cano A, Pérez-Moreno MA, Rodrigo I (2000) The transcription factor snail controls epithelial–mesenchymal transitions by repressing E-cadherin expression. Nature cell … 2.

8. Villarejo A, Cortés-Cabrera A, Molina-Ortíz P, Portillo F, Cano A (2014) Differential role of Snail1 and Snail2 zinc fingers in E-cadherin repression and epithelial to mesenchymal transition. The Journal of biological chemistry 289: 930-941.

9. Bracken CP, Gregory Pa, Kolesnikoff N, Bert AG, Wang J, et al. (2008) A double-negative feedback loop between ZEB1-SIP1 and the microRNA-200 family regulates epithelial-mesenchymal transition. Cancer research 68: 7846-7854.

10. Burk U, Schubert J, Wellner U, Schmalhofer O, Vincan E, et al. (2008) A reciprocal repression between ZEB1 and members of the miR-200 family promotes EMT and invasion in cancer cells. EMBO reports 9: 582-589.

11. Ahn YH, Gibbons DL (2012) ZEB1 drives prometastatic actin cytoskeletal remodeling by downregulating miR-34a expression. The Journal of Clinical Investigation 122.

12. Eger A, Aigner K, Sonderegger S, Dampier B, Oehler S, et al. (2005) DeltaEF1 is a transcriptional repressor of E-cadherin and regulates epithelial plasticity in breast cancer cells. Oncogene 24: 2375-2385.

13. Liu Y, El-Naggar S, Darling DS, Higashi Y, Dean DC (2008) Zeb1 links epithelial-mesenchymal transition and cellular senescence. Development 135: 579-588.

14. Wang B, Koh P, Winbanks C, Coughlan MT, McClelland A, et al. (2011) miR-200a Prevents renal fibrogenesis through repression of TGF-β2 expression. Diabetes 60: 280-287.

15. Gregory Pa, Bracken CP, Smith E, Bert AG, Wright Ja, et al. (2011) An autocrine TGF-beta/ZEB/miR-200 signaling network regulates establishment and maintenance of epithelial-mesenchymal transition. Molecular biology of the cell 22: 1686-1698.

16. Watanabe K, Villarreal-Ponce A, Sun P, Salmans ML, Fallahi M, et al. (2014) Mammary morphogenesis and regeneration require the inhibition of EMT at terminal end buds by Ovol2 transcriptional repressor. Developmental cell 29: 59-74.

17. Lee B, Villarreal-ponce A, Fallahi M, Ovadia J, Sun P, et al. (2014) Transcriptional Mechanisms Link Epithelial Plasticity to Adhesion and Differentiation of Epidermal Progenitor Cells. Developmental Cell 29: 47-58.

18. Roca H, Hernandez J, Weidner S, McEachin RC, Fuller D, et al. (2013) Transcription factors OVOL1 and OVOL2 induce the mesenchymal to epithelial transition in human cancer. PloS one 8: e76773-e76773.
