# Supplementary material for: An Ovol2-Zeb1 Mutual Inhibitory Circuit Governs Bidirectional and Multi-step Transition between Epithelial and Mesenchymal States
Source: PLoS Comput Biol. 2015 Nov 10;11(11):e1004569. doi: 10.1371/journal.pcbi.1004569 (PMC4640575; doi:10.1371/journal.pcbi.1004569)
Supplement: S2 Table — (DOCX) [file pcbi.1004569.s003.docx]

**Table S2 List of basal parameter values**

| **Name** | **Description** | **Value** |
| --- | --- | --- |
| **$J1_{200}$** | **$\text{Michaelis constant of Snail-dependent inhibition of miR-200 production}$** | **$\text{3 μΜ}$** |
| **$J2_{200}$** | **$\text{Michaelis constant of Zeb1-dependent inhibition of miR-200 production}$** | **$\text{0.2 μΜ}$** |
| **$J1_{34}$** | **$\text{Michaelis constant of Snail-dependent inhibition of miR-34 production}$** | **$\text{0.15 μΜ}$** |
| **$J2_{34}$** | **$\text{Michaelis constant of Zeb1-dependent inhibition of miR-34 production}$** | **$\text{0.35μΜ}$** |
| **$J_{O}$** | **$\text{Michaelis constant of Zeb1-dependent inhibition of Ovol2 production}$** | **$\text{0.9 μΜ}$** |
| **$J0_{snail}$** | **$\text{Michaelis constant of TGF-β-dependent }Snail1\text{ mRNA transcription}$** | **$\text{0.6μΜ}$** |
| **$J1_{snail}$** | **$\text{Michaelis constant of Ovol2-dependent inhibition of }Snail1\text{ mRNA transcription}$** | **$\text{0.5 μΜ}$** |
| **$J2_{snail}$** | **$\text{Michaelis constant of Snail self-inhibition}$** | **$\text{1.8 μΜ}$** |
| **$J1_{E}$** | **$\text{Michaelis constant of Snail-dependent inhibition of Ecad production}$** | **$\text{0.1 μΜ}$** |
| **$J2_{E}$** | **$\text{Michaelis constant of Zeb1-dependent inhibition of Ecad production}$** | **$\text{0.3 μΜ}$** |
| **$J1_{V}$** | **$\text{Michaelis constant of Snail-dependent Vim production}$** | **$\text{0.4 μΜ}$** |
| **$J2_{V}$** | **$\text{Michaelis constant of Zeb1-dependent Vim production}$** | **$\text{0.4 μΜ}$** |
| **$J3_{V}$** | **$\text{Michaelis constant of Ovol2-dependent inhibition of Vim production}$** | **$\text{2 μΜ}$** |
| **$J1_{zeb}$** | **$\text{Michaelis constant of Snail-dependent }Zeb1\text{ transcription}$** | **$\text{3.5 μΜ}$** |
| **$J2_{zeb}$** | **$\text{Michaelis constant of Ovol2-dependent inhibition of }Zeb1\text{ transcription}$** | **$\text{0.9 μΜ}$** |
| **$K_{1}$** | **$\text{Binding/unbinding rate ratio for the first miR-200 molecule and }Zeb1\text{ mRNA}$** | **$\text{1.0/μΜ}$** |
| **$K_{2}$** | **$\text{Binding/unbinding rate ratio for the second miR-200 molecule and }Zeb1\text{ mRNA}$** | **$\text{1.0/μΜ}$** |
| **$K_{3}$** | **$\text{Binding/unbinding rate ratio for the third miR-200 molecule and }Zeb1\text{ mRNA}$** | **$\text{1.0/μΜ}$** |
| **$K_{4}$** | **$\text{Binding/unbinding rate ratio for the fourth miR-200 molecule and }Zeb1\text{ mRNA}$** | **$\text{1.0/μΜ}$** |
| **$K_{5}$** | **$\text{Binding/unbinding rate ratio for the fifth miR-200 molecule and }Zeb1\text{ mRNA}$** | **$\text{1.0/μΜ}$** |
| **$K_{TR}$** | **$\text{Binding/unbinding rate ratio for miR-200 and }TGF\text{-}\beta\text{ mRNA}$** | **$\text{20/μΜ}$** |
| **$K_{SR}$** | **$\text{Binding/unbinding rate ratio for miR-34 molecule and }Snail1\text{ mRNA}$** | **$\text{100/μΜ}$** |
| **$TGF0$** | **$\text{Concentration of exogenous TGF-β}$** | $\text{0 μM}$ |
| **$Tk$** | **$\text{Timescale constant for all miRNA-mRNA binding/unbinding events}$** | **$1000$** |
| **$k0_{O}$** | **$\text{Basal production rate of Ovol2}$** | **$\text{0.35 μM/hr}$** |
| **$k0_{200}$** | **$\text{Basal production rate of miR-200}$** | **$\text{0.0002 μM/hr}$** |
| **$k0_{34}$** | **$\text{Basal production rate of miR-34}$** | **$\text{0.001 μM/hr}$** |
| **$k0_{snail}$** | **$\text{Basal production rate of }Snail1\text{ mRNA}$** | **$\text{0.0005 μM/hr}$** |
| **$k0_{zeb}$** | **$\text{Basal production rate of }Zeb1\text{ mRNA}$** | **$\text{0.003 μM/hr}$** |
| **$k0_{TGF}$** | **$\text{Basal production rate of TGF-β}$** | **$\text{1.1 μΜ/hr}$** |
| **$k0_{E}$** | **$\text{Basal production rate of Ecad}$** | **$\text{5 μΜ/hr}$** |
| **$k0_{V}$** | **$\text{Basal production rate of Vim}$** | **$\text{5 μΜ/hr}$** |
| **$k_{E1}$** | **$\text{Production rate 1 of Ecad}$** | **$\text{15 μΜ/hr}$** |
| **$k_{E2}$** | $\text{Production rate 2 of Ecad}$ | $\text{5 μΜ/hr}$ |
| $k_{V1}$ | $\text{Production rate 1 of Vim}$ | $\text{2 μΜ/hr}$ |
| $k_{V2}$ | $\text{Production rate 2 of Vim}$ | $\text{5 μΜ/hr}$ |
| $k_{O}$ | $\text{Production rate of Ovol2}$ | $\text{1.2 μΜ/hr}$ |
| $k_{200}$ | $\text{Production rate of miR-200}$ | $\text{0.02 μΜ/hr}$ |
| $k_{34}$ | $\text{Production rate of miR-34}$ | $\text{0.01 μΜ/hr}$ |
| $k_{snail}$ | $\text{Transcription rate of }Snail1\text{ mRNA}$ | $\text{0.05 μΜ/hr}$ |
| $k_{tgf}$ | $\text{Transcription rate of TGF-β}$ | $\text{0.05 μΜ/hr}$ |
| $k_{zeb}$ | $\text{Transcription rate of }Zeb1\text{ mRNA}$ | $\text{0.06 μΜ/hr}$ |
| $k_{TGF}$ | $\text{Production rate of TGF-β protein}$ | $\text{1.5 μΜ/hr}$ |
| $k_{SNAIL}$ | $\text{Production rate of Snail protein}$ | $\text{16 μΜ/hr}$ |
| $k_{ZEB}$ | $\text{Production rate of Zeb1 protein}$ | $\text{16 μΜ/hr}$ |
| $kd_{ZR_{1}}$ | $\text{Degradation rate for complex of 1 }Zeb1\text{ mRNA and 1 miR-200 molecules}$ | $\text{0.5 /hr}$ |
| $kd_{ZR_{2}}$ | $\text{Degradation rate for complex of 1 }Zeb1\text{ mRNA and 2 miR-200 molecules}$ | $\text{0.5 /hr}$ |
| $kd_{ZR_{3}}$ | $\text{Degradation rate for complex of 1 }Zeb1\text{ mRNA and 3 miR-200 molecules}$ | $\text{0.5 /hr}$ |
| $kd_{ZR_{4}}$ | $\text{Degradation rate for complex of 1 }Zeb1\text{ mRNA and 4 miR-200 molecules}$ | $\text{0.5 /hr}$ |
| $kd_{ZR_{5}}$ | $\text{Degradation rate for complex of 1 }Zeb1\text{ mRNA and 5 miR-200 molecules}$ | $\text{0.5 /hr}$ |
| $kd_{O}$ | $\text{Degradation rate of Ovol2 }$ | $\text{1.0 /hr}$ |
| $kd_{200}$ | $\text{Degradation rate of miR-200}$ | $\text{0.035 /hr}$ |
| $kd_{34}$ | $\text{Degradation rate of miR-34}$ | $\text{0.035 /hr}$ |
| $kd_{SR}$ | $\text{Degradation rate of complex }Snail1\text{ mRNA and miR-34}$ | $\text{0.9 /hr}$ |
| $kd_{E}$ | $\text{Degradation rate of Ecad}$ | $\text{0.05 /hr}$ |
| $kd_{V}$ | $\text{Degradation rate of Vim}$ | $\text{0.05 /hr}$ |
| $kd_{snail}$ | $\text{Degradation rate of }Snail1\text{ mRNA}$ | $\text{0.09 /hr}$ |
| $kd_{tgf}$ | $\text{Degradation rate of }TGF\text{-}\beta\text{ mRNA}$ | $\text{0.1 /hr}$ |
| $kd_{TR}$ | $\text{Degradation rate of of complex }TGF\text{-}\beta\text{ mRNA and miR-200}$ | $\text{1.0 /hr}$ |
| $kd_{zeb}$ | $\text{Degradation rate of }Zeb1\text{ mRNA}$ | $\text{0.10 /hr}$ |
| $kd_{SNAIL}$ | $\text{Degradation rate of Snail protein}$ | $\text{1.6 /hr}$ |
| $kd_{TGF}$ | $\text{Degradation rate of TGF-β protein}$ | $\text{0.9/hr}$ |
| $kd_{ZEB}$ | $\text{Degradation rate of Zeb1 protein}$ | $\text{1.66/hr}$ |
| $n1_{200}$ | $\text{Hill exponent of Snail-dependent inhibition of miR-200 production}$ | $3$ |
| $n2_{200}$ | $\text{Hill exponent of Zeb1-dependent inhibition of miR-200 production}$ | $2$ |
| $n1_{34}$ | $\text{Hill exponent of Snail-dependent inhibition of miR-34 production}$ | $2$ |
| $n2_{34}$ |  |  |
|  |  |  |
|  |  |  |
|  |  |  |
|  |  |  |
|  |  |  |
|  |  |  |
|  |  |  |
|  |  |  |
|  |  |  |
|  |  |  |
|  |  |  |
|  |  |  |
|  |  |  |
|  |  |  |
|  |  |  |
|  |  |  |
