# Supplementary figures and images for: An Ovol2-Zeb1 Mutual Inhibitory Circuit Governs Bidirectional and Multi-step Transition between Epithelial and Mesenchymal States
Source: PLoS Comput Biol. 2015 Nov 10;11(11):e1004569. doi: 10.1371/journal.pcbi.1004569 (PMC4640575; doi:10.1371/journal.pcbi.1004569)

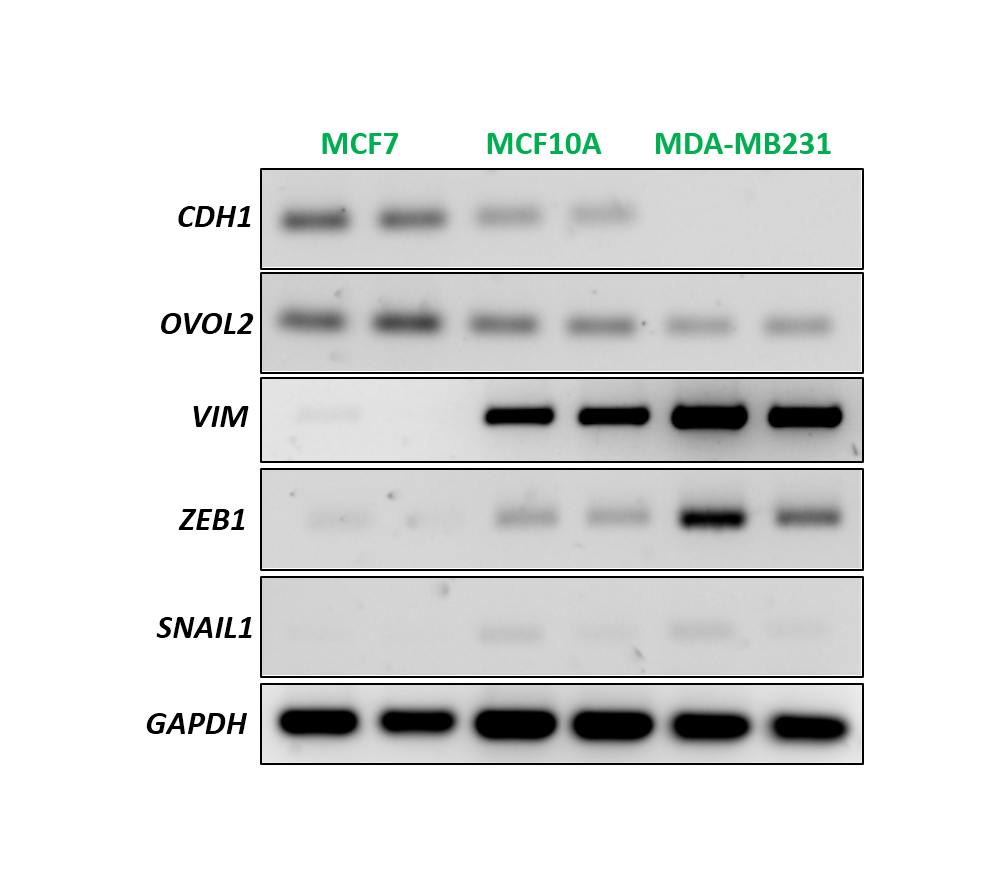

Supplement: S1 Fig — RT-semi-quantitative PCR analysis of the indicated genes in three human breast cell lines. Two biological replicates were performed for each gene in each type of cell line. (TIF) [file pcbi.1004569.s004.tif]

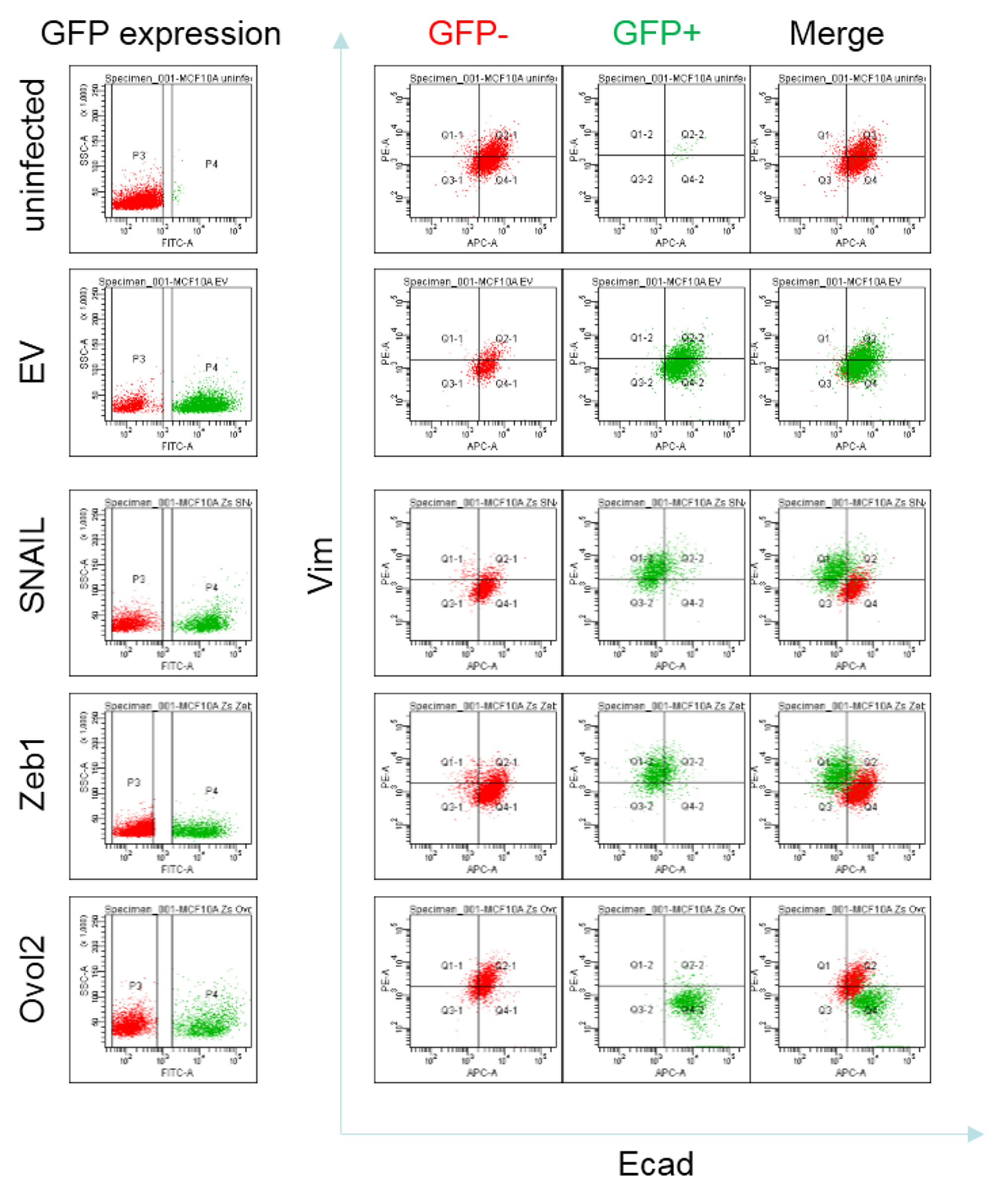

Supplement: S2 Fig — Successfully infected population (GFP-positive) can be distinguished from the uninfected population (GFP-negative) by GFP fluorescence (left panels). Ecad/Vim profiles are visualized separately for GFP-positive and–negative populations. Analysis was performed on MCF10A cells five days after infection. Note that GFP-negative population serves as an internal control. Only GFP-positive populations were analyzed in the experiments for main figures. (TIF) [file pcbi.1004569.s005.tif]

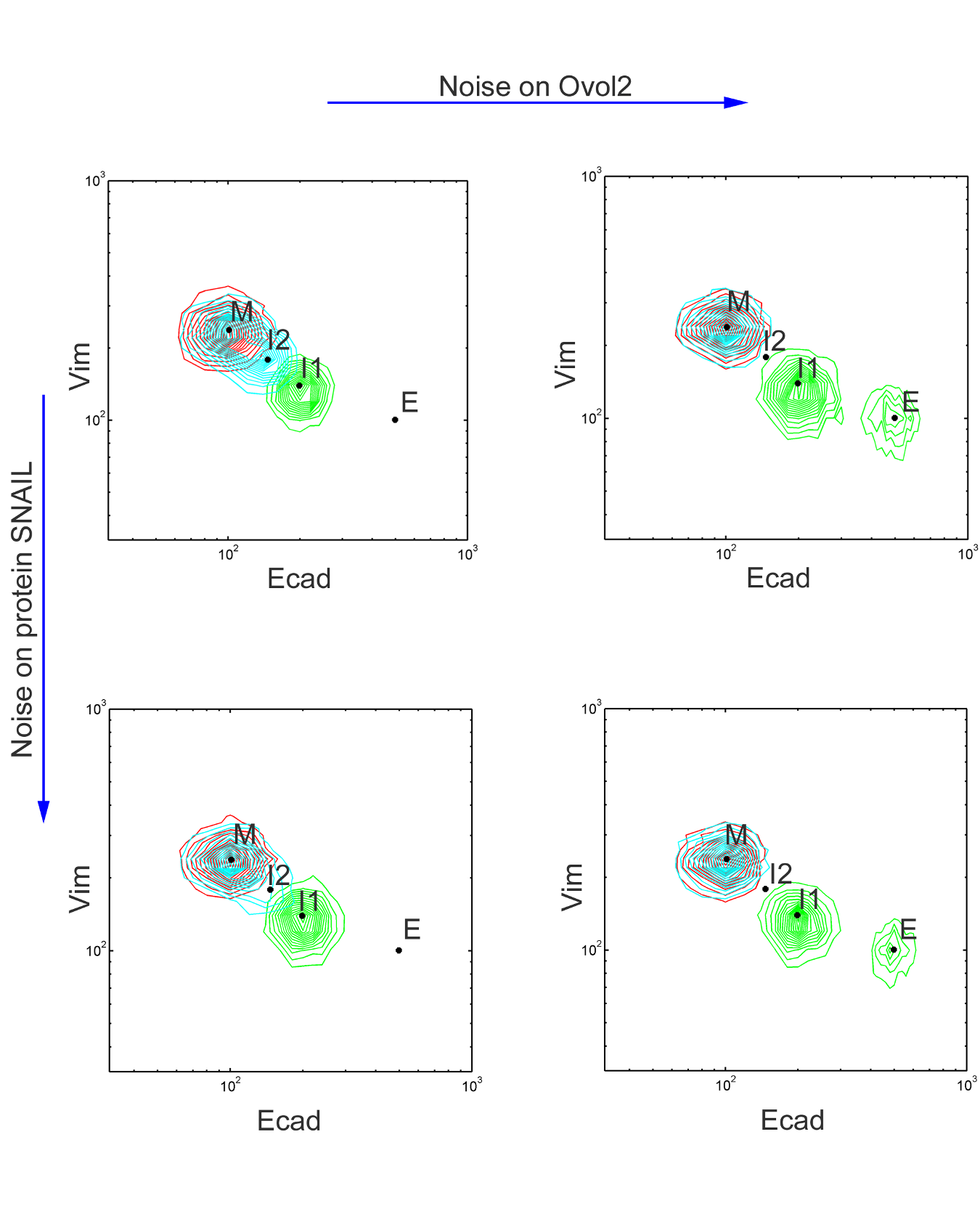

Supplement: S3 Fig — Stochastic simulation (started at I1 state) for a population of 2000 cells at three concentration of TGF-β. Green: no TGF-β (I1 state). Red: high (10 units) TGF-β concentration (M state). Cyan: intermediate (2.5 units) TGF-β concentration (a mixture of I2 and M populations can be obtained at the low-noise condition). At high TGF-β concentration, the system is monostable at M state. At intermediate TGF-β concentration, the system is bistable at M or I2 state. See Fig 2B. (TIF) [file pcbi.1004569.s006.tif]

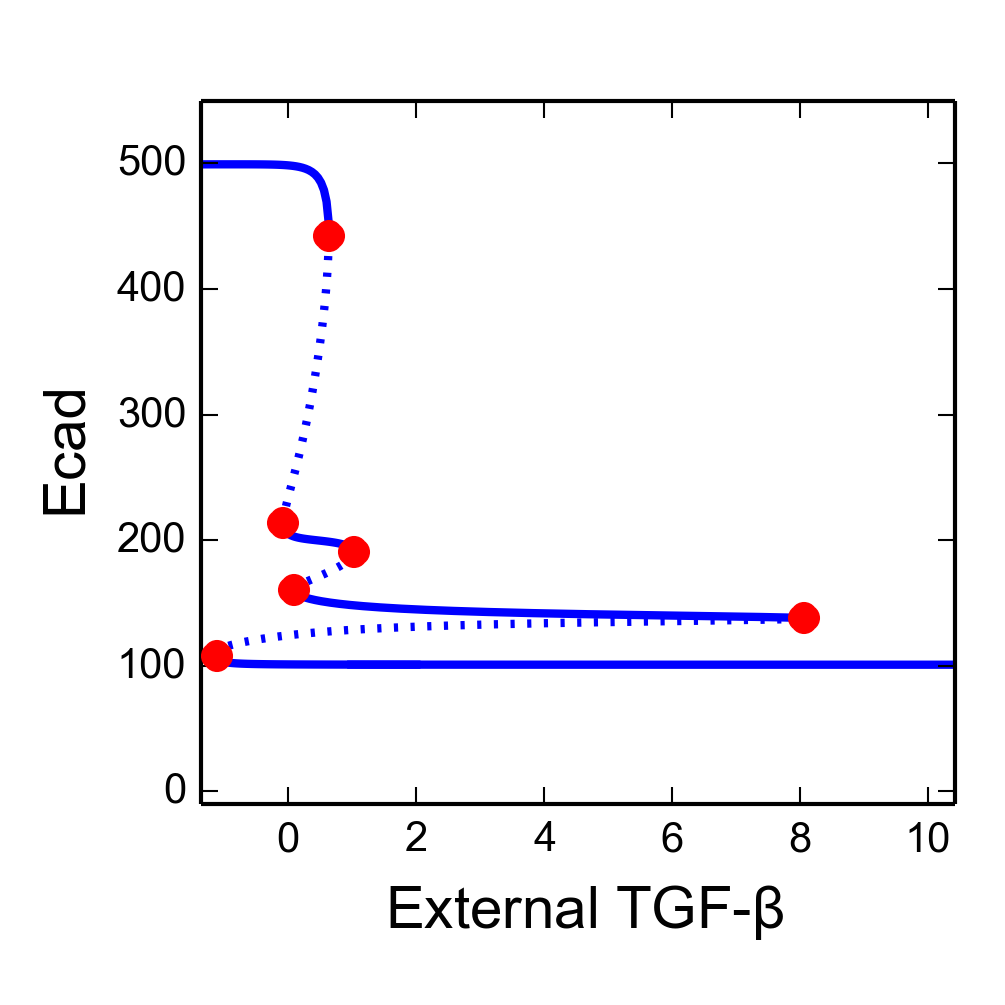

Supplement: S4 Fig — Solid curve: stable steady state. Dashed curve: unstable steady state. Red dots: saddle-node bifurcation points used for computing curves in Fig 6A. (TIF) [file pcbi.1004569.s007.tif]

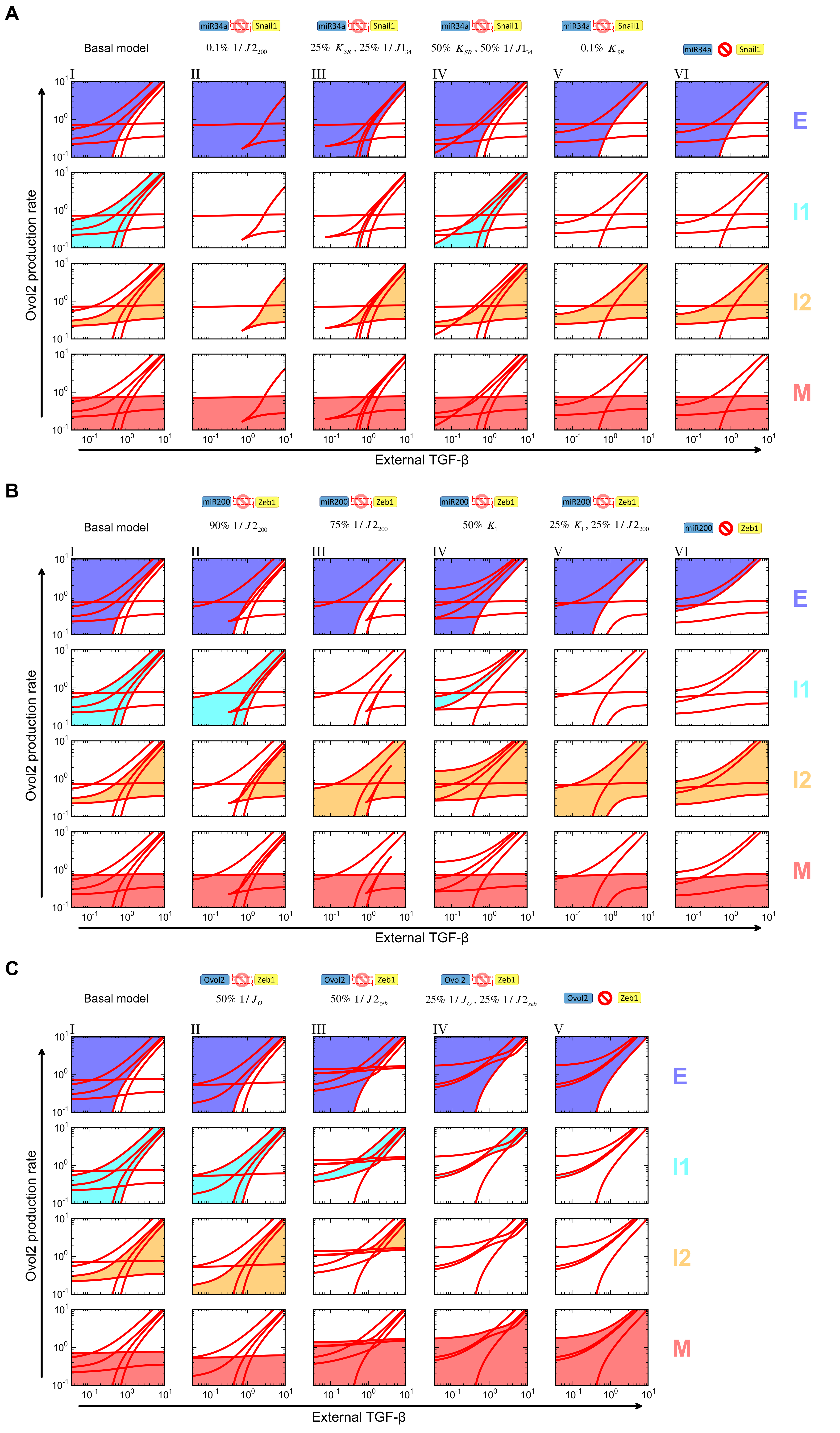

Supplement: S5 Fig — Shown are comparisons of the basal model (leftmost column), complete blockage (rightmost column), and partial blockage (middle columns) of miR34a-Snail (A), miR200-Zeb1 (B) and Ovol2-Zeb1 (C) mutual inhibition loops on the four phenotypes. Each subplot is a two-parameter bifurcation diagram similar to Fig 6A. Subplots in each column highlight the various phenotypes in one condition. Shaded areas are highlighted phenotypes. Colors of the shading correspond to the colored labels on the right. (TIF) [file pcbi.1004569.s008.tif]

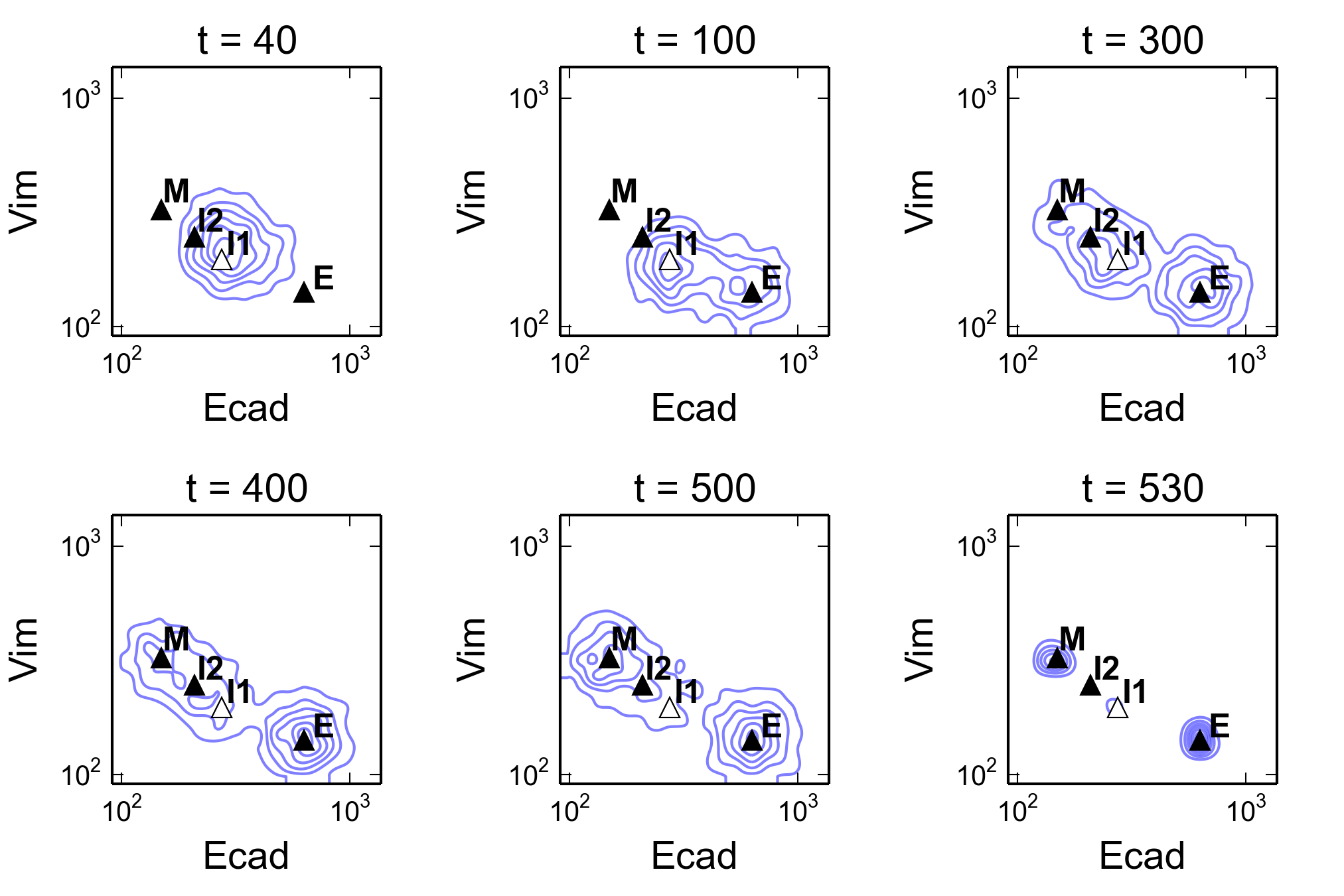

Supplement: S6 Fig — Stochastic simulations for a population of 2000 cells. The basal parameter set and initial condition at I1 used (as in Fig 9). TGF-β concentration was raised from 0 to 0.5 at t = 100. White noise terms were set to zero at t = 500. (TIF) [file pcbi.1004569.s009.tif]
